# Activity-dependent interdomain dynamics of matrix metalloprotease-1 on fibrin

**DOI:** 10.1038/s41598-020-77699-3

**Published:** 2020-11-26

**Authors:** Lokender Kumar, Joan Planas-Iglesias, Chase Harms, Sumaer Kamboj, Derek Wright, Judith Klein-Seetharaman, Susanta K. Sarkar

**Affiliations:** 1 Department of Physics, Colorado School of Mines, 1500 Illinois Street, Golden, CO 80401 USA; 2 Warwick Medical School, University of Warwick, Coventry, CV4 7AL UK; 3 Loschmidt Laboratories, Department of Experimental Biology, Faculty of Science, Masaryk University, Kamenice 5/A13, 625 00 Brno, Czech Republic; 4 Department of Chemistry, Colorado School of Mines, 1500 Illinois Street, Golden, CO 80401 USA

**Keywords:** Biophysics, Molecular biophysics, Molecular conformation

## Abstract

The roles of protein conformational dynamics and allostery in function are well-known. However, the roles that interdomain dynamics have in function are not entirely understood. We used matrix metalloprotease-1 (MMP1) as a model system to study the relationship between interdomain dynamics and activity because MMP1 has diverse substrates. Here we focus on fibrin, the primary component of a blood clot. Water-soluble fibrinogen, following cleavage by thrombin, self-polymerize to form water-insoluble fibrin. We studied the interdomain dynamics of MMP1 on fibrin without crosslinks using single-molecule Forster Resonance Energy Transfer (smFRET). We observed that the distance between the catalytic and hemopexin domains of MMP1 increases or decreases as the MMP1 activity increases or decreases, respectively. We modulated the activity using (1) an active site mutant (E219Q) of MMP1, (2) MMP9, another member of the MMP family that increases the activity of MMP1, and (3) tetracycline, an inhibitor of MMP1. We fitted the histograms of smFRET values to a sum of two Gaussians and the autocorrelations to an exponential and power law. We modeled the dynamics as a two-state Poisson process and calculated the kinetic rates from the histograms and autocorrelations. Activity-dependent interdomain dynamics may enable allosteric control of the MMP1 function.

## Introduction

**Figure 1:**
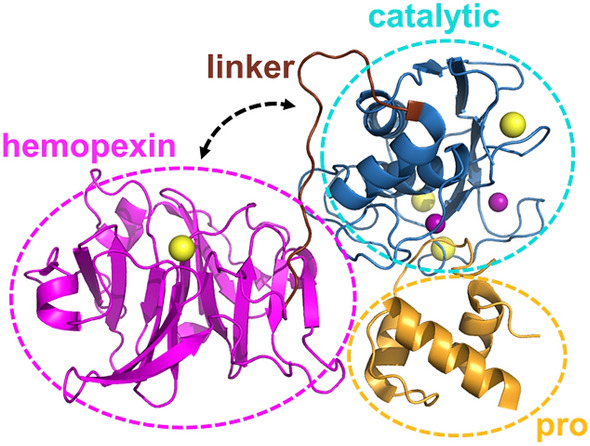
MMP1 structure (PDB ID: ISU3). The hemopexin domain is connected to the catalytic domain by a flexible linker. The pro domain is cleaved off to activate MMPs for catalysis. Yellow and purple spheres represent the van der Waals radii of the calcium and zinc atoms, respectively. The pro domain, the catalytic domain, the linker, and the hemopexin domains are roughly defined by the ranges of residues D32-Q99, F100-Y260, G261-C278, and D279-C466, respectively.

Understanding the relationship between the structure and dynamics of a protein is crucial for understanding its function^1^. Researchers have shown the correlation between the function and intra-domain dynamics within a protein domain at different hierarchies of timescales^2–4^. However, the roles of interdomain dynamics in function are not entirely understood^5,6^. Matrix metalloproteases (MMPs) are suitable for investigating the functional relationship between interdomain dynamics and activity because the sequence of the catalytic domain remains conserved across the 23-member family^7^. The differences in activities among MMPs likely originate from the hemopexin domain with significant variations in the sequence^7^. The hemopexin domain seems to influence substrate/ligand specificity and activation/inhibition of various MMPs^8^. Also, prior research has reported the regulation of MMP1 catalytic activity by the hemopexin domain^9^. MMPs can degrade numerous proteins^10^, including collagen, the primary component of the extracellular matrix (ECM) that provides a scaffold for cells to maintain tissue integrity^11^. MMPs are calcium- and zinc-dependent enzymes produced by cells in pro forms, i.e., they need to be activated by cleaving off the pro domain for activity^11,12^. MMP1, a collagenase that degrades triple-helical type-1 collagen, stands out in the 23-member MMP family because it has crystal structures available^13^. Also, MMP1 itself is a broad-spectrum protease, an attribute that we previously used to degrade *E. coli* proteins and purify recombinant MMP1 using a protease-based method^14^. Extensive studies of MMP1 interacting with collagen monomers have revealed significant insights into the MMP1 activity, including the roles of conformational dynamics^9,15–22^. MMP1 consists of a catalytic domain that degrades substrates, a hemopexin domain that helps MMPs bind to the substrates, and a linker that mediates communications between the two domains (Fig. 1). We recently showed that the interdomain dynamics of MMP1 on type-1 collagen fibrils correlate with activity, and the two domains can allosterically communicate via the linker region and substrate^23^. Since MMP1 is promiscuous and interacts with diverse substrates^10^, we investigated whether the insights into triple-helical collagen fibril degradation applies to triple-helical fibrin degradation.

This paper focuses on MMP1 interdomain dynamics and fibrinolytic activity, the primary component of a blood clot. The motivation behind our studies on fibrin stems from the triple-helical structural similarities between collagen and fibrin. Fibrin monomers have three right-handed chains forming a left-handed structure^24^. In contrast, collagen monomers have three left-handed chains forming a right-handed structure^25^. As such, we expect to define the similarities and differences of the structure-dynamics-function relationship of MMP1 on these two substrates to gain more insights. An understanding of MMP1-fibrin interactions has potential implications in fibrinolytic pathways. Commonly, tissue plasminogen activator (tPA) remains the standard treatment for ischemic stroke to degrade blood clot^26^. Plasminogen becomes plasmin after cleavage by tPA and can degrade blood clots^27^. However, the observation of clot degradation in plasminogen-deleted animals suggests alternative fibrinolytic pathways^28^. In this context, MMPs are potential alternatives in the fibrinolytic system because MMPs are in the blood in pro^29,30^ and activated^31–34^ forms. There are reports of fibrinolytic activity of MMP3^35^, MMP9^36^, and MMP14^37^, but the field is relatively unexplored^38^. Prior research has reported that MMP1 does not have significant fibrinolytic activity^35^, which contrasts with our results presented in this paper.

Here we show that MMP1 interdomain dynamics on fibrin are activity-dependent and modulated by an enhancer or an inhibitor of MMP activity. We calculated the distance between S142 in the catalytic domain and S366 in the hemopexin domain of MMP1 using smFRET measurements. The area-normalized histograms of smFRET values represent conformations' distributions, whereas the normalized autocorrelations represent correlations between structures at different time points. From the histograms, we defined two MMP1 conformations with (1) interdomain distance of ~ 4.5 nm and (2) interdomain distance of ~ 5.4 nm as the closed and open states, respectively. A comparison of active and catalytically inactive mutant E219Q^14,23,39–41^ suggests that the open conformation with well-separated domains of MMP1 is functionally essential for degrading fibrin. We recently showed that the open conformation of MMP1 is also necessary for degrading collagen^23^, another triple-helical substrate like fibrin. For collagen, the open conformation's functional importance resolved the debate about the open and closed conformations^23^. In other words, the open conformation is functionally essential for both collagen and fibrin. The open conformations appear more in the presence of an activity enhancer (MMP9) and less with an inhibitor (tetracycline). From autocorrelations, we learned that interdomain dynamics are not entirely random and have exponentially-distributed correlations. We fitted a sum of two Gaussians to the histograms and an exponential to the autocorrelations. We modeled the dynamics as a two-state process, where the locations of two states are the centers of Gaussian fits, and the sum of two kinetic rates between the two states is the decay rate of exponential fits. Anisotropic Network Modeling (ANM) of MMP1 dynamics revealed that a larger separation between the two domains (open conformation) often accompanies a larger catalytic pocket opening between N171 and T230. A larger catalytic pocket opening, in turn, enables closer proximity to the three chains of fibrin. ANM simulations further revealed that the α-chain of fibrin is closest to the MMP1 active site, whereas the γ-chain is furthest. SDS PAGE of ensemble fibrin degradation confirmed that MMP1 cleaves the α-chain first, followed by the β- and γ-chains. Single molecule experiments, stochastic simulations, ANM simulations, and ensemble assays provide an integrative approach to investigating interdomain dynamics' functional roles. This approach applies to other biochemical processes where water-soluble enzymes interact with water-insoluble substrates.

## Results and discussion

### Single-molecule measurement of MMP1 interdomain dynamics on fibrin without crosslinks

Water-soluble fibrinogen, upon limited cleavage by thrombin, self-polymerize into water-insoluble fibrin without crosslinks. The addition of factor XIII and CaCl_2_ creates fibrin with crosslinks. We used fibrin without crosslinks for single molecule measurements because we used a fibrin crystal structure without crosslinks for simulations. Besides, fibrin without crosslinks is optically clearer than fibrin with crosslinks, facilitating imaging with less background. We measured smFRET between two dyes attached to the catalytic and hemopexin domains of MMP1 to measure dynamics at the single molecule level. We mutated S142 in the catalytic domain and S366 in the hemopexin domain of MMP1 to cysteines for labeling. The distances between S142 and S366 are ~ 5.4 nm and ~ 4.5 nm for the open and closed conformations of MMP1, respectively. We selected a pair of dyes, Alexa555 and Alexa647, because the Forster radius between the two fluorophores is 5.1 nm. The FRET efficiency is 50% when the distance between the fluorophores is equal to the Forster radius. More importantly, FRET is most sensitive to any distance change around the Forster radius.

**Figure 2:**
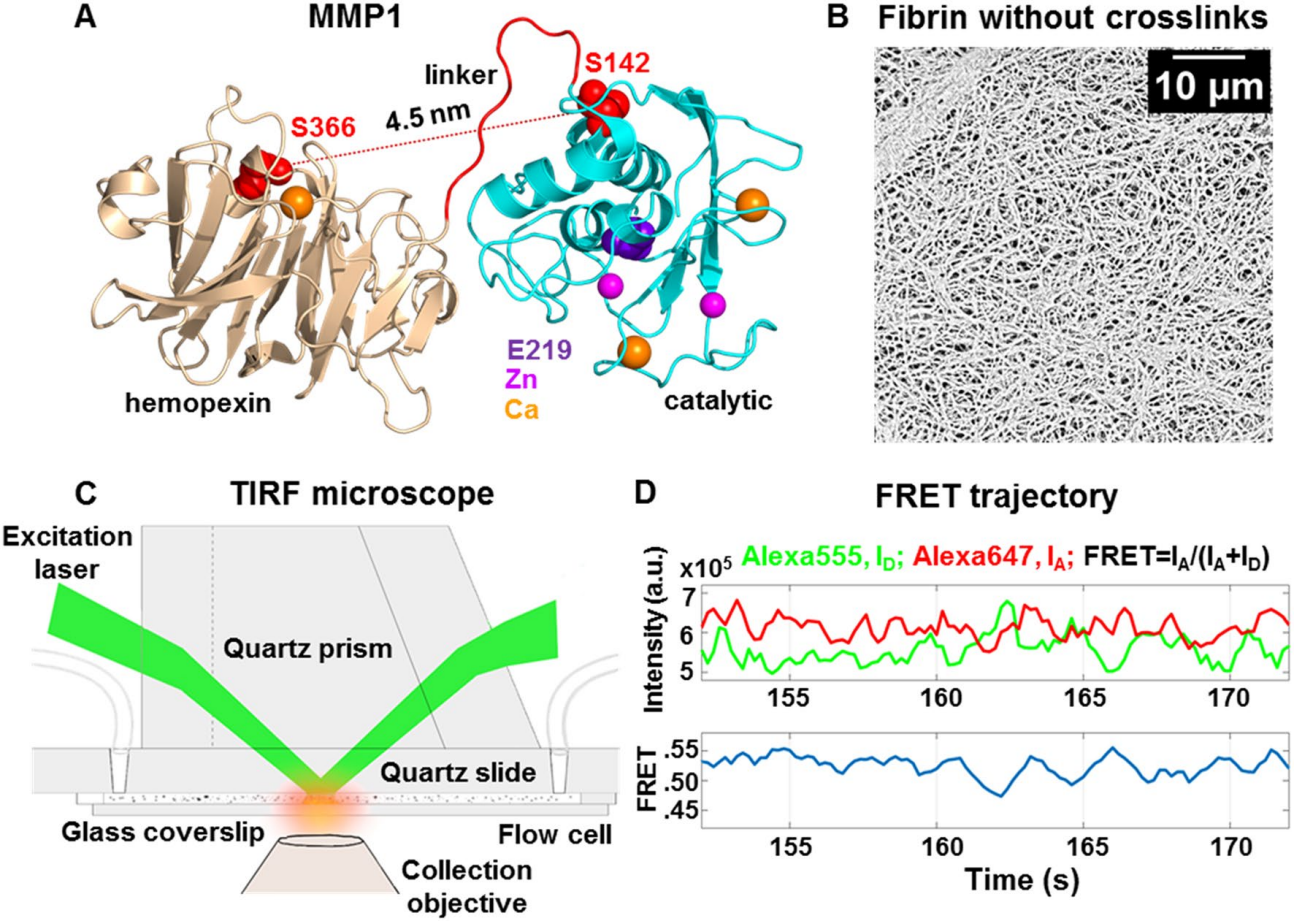
Single-molecule measurement of MMP1 dynamics on fibrin without crosslinks. (**A**) Crystal structure of MMP1 (PDB ID: 1SU3). Mutations of S142 and S366 to cysteines enables attaching Alexa555 and Alexa647 dyes. (**B**) Scanning Electron Microscope (SEM) images of fibrin without crosslinks. (**C**) Schematics of the TIRF microscope used for measuring MMP1 interdomain dynamics on fibrin. (**D**) Emission intensities of the two dyes at 22 °C with a 100 ms time resolution. (top panel) Low FRET conformations lead to high Alexa555 emission, whereas High FRET conformations lead to Low Alexa555 emission. Anticorrelated Alexa647 and Alexa555 emissions, I_A_ and I_D_, respectively; (bottom panel) Calculated smFRET trajectory to show MMP1 interdomain dynamics as a function of time.

**Figure 3:**
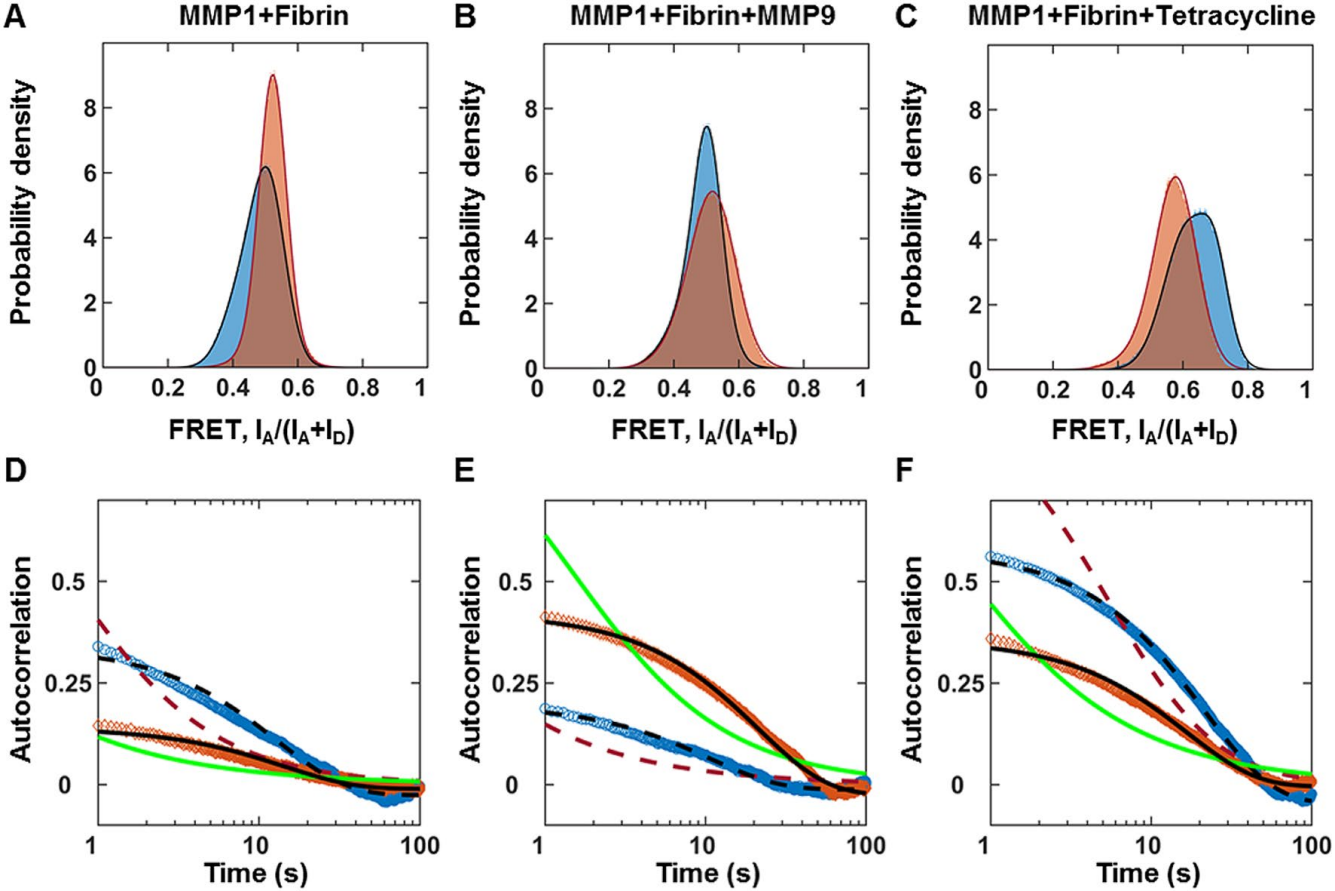
Interdomain dynamics of MMP1 on fibrin correlates with activity. More than 300,000 smFRET values at 22 °C with a 100 ms time resolution for each condition are used to create area-normalized histograms of MMP1 interdomain distance (bin size = 0.005). (**A**) Histograms without ligand, (**B**) Histograms in the presence of MMP9 (an enhancer), and (**C**) Histograms in the presence of tetracycline (an inhibitor) for active (blue) and active site mutant (orange) MMP1. Histograms are fitted to a sum of two Gaussians (active: solid blue line; active site mutant: solid red line). Autocorrelations of MMP1 interdomain distance are calculated from the time series of smFRET values. (**D**) Autocorrelations without ligand, (**E**) Autocorrelations in the presence of MMP9, and (**F**) Autocorrelations in the presence of tetracycline for active MMP1 (blue) and active site mutant of MMP1 (orange). Autocorrelations are fitted to exponentials and power laws (exponential fit to active: dashed black line; power law fit to active: dashed red line; exponential fit to active site mutant: solid black line; power law fit to active site mutant: solid green line). The error bars in the histograms and autocorrelations represent the square roots of the bin counts and the standard errors of the mean (sem) and are too small to be seen. The supplementary information contains the fit equations and the best-fit parameters for histograms and autocorrelations (Table S1). We approximated smFRET efficiency by I_A_/( I_A_ + I_D_)^49^ and has no unit.

Single molecule measurements suffer^42^ from labeling^43^, solution conditions^44^, and properties of fluorophores^45–48^. The stochastic nature of labeling CYS142 and CYS366 can also be a problem discussed in our previous publication^23^. Since active MMP1 and active site mutant of MMP1 would be affected equally by these complications, we can distinguish the effects due to activity. Note that the amino acid E219^14^ is the same as E200^13^, which differs because we included 19 residues of the pro domain. In a previous publication, we showed that the specific activities of labeled and unlabeled MMP1 were not affected by labeling^23^. A flexible linker connects the catalytic and hemopexin domains of MMP1 (Fig. 2A). Figure 2B shows fibrin without crosslinks. We measured MMP1 interdomain dynamics on a thin layer of water-insoluble fibrin in a flow cell using a Total Internal Reflection Fluorescence (TIRF) microscope (Fig. 2C). Figure 2D shows one smFRET trajectory. For each condition, we collected more than 300,000 smFRET values for analysis of MMP1 interdomain dynamics on fibrin (Fig. 3).

### MMP1 interdomain dynamics on fibrin correlate with activity

Each value of smFRET represents a conformation of MMP1. The histograms of smFRET values measuring interdomain distances (Fig. 3A) for active MMP1 and active site mutant of MMP1 suggest that low FRET conformations, where the two domains are well-separated, occur more for active MMP1 compared to the active site mutant. See supplementary information for the definition of low FRET and high FRET states. The prevalence of low FRET conformations for active MMP1 suggests that the two domains need to move away from each other for performing catalytic activity on fibrin. Previously, we observed the same effects for MMP1 function on type-1 collagen^23^. Collagen degradation by MMP1 is enhanced by MMP9 and inhibited by tetracycline. One possible explanation of MMP1 inhibition by tetracycline depends on how tetracycline binds to MMP1. Tetracycline primarily binds to MMP1 at two places: one at the active site of MMP1 and the other between two domains. The docking pose between the two domains has the lowest binding energy, where tetracycline binds to the two MMP1 domains via four hydrogen bonds and prevents interdomain dynamics of MMP1^23^. The mechanism of MMP1 activity enhancement by MMP9 is not clear yet and needs further studies. The justification for using tetracycline is that doxycycline hyclate, a tetracycline derivative, is a well-known MMP inhibitor for therapeutics^50^. MMP9, another MMP that cannot degrade type-1 collagen with intact triple-helical structure, can degrade denatured collagen^51^. MMP9 is found in blood^29,30^, increases in many diseases^52^, forms a complex with MMP1^53^, can degrade fibrin^54^, and enhances degradation by MMP1^23^. For collagen, we found that low FRET conformations appear more for MMP9 and less for tetracycline, consistent with the enhancement and inhibition of MMP1 activity. To test whether or not MMP1 follows similar interdomain dynamics when bound to fibrin, we performed smFRET measurements in the presence of MMP9 and tetracycline. In the presence of tetracycline, low FRET conformations of MMP1 on fibrin significantly disappear on fibrin (Fig. 3C), as observed on type-1 collagen. Surprisingly, in the presence of MMP9, the MMP1 interdomain dynamics on fibrin (Fig. 3B) show more high FRET states in contrast to the occurrence of more low FRET states on collagen. To determine how a conformation at one time point correlates with a conformation at another time point, we calculated the autocorrelations of conformations (Fig. 3D–F). Without any ligand, the correlation of dynamics on fibrin (Fig. 3D) is higher for active MMP1 at shorter times, similar to previously reported dynamics on collagen. However, the correlations in the presence of MMP9 and tetracycline show opposite orders on fibrin (Fig. 3E,F) and collagen^23^. The correlated motions indicate a decrease in conformational entropy. They can affect kinetics and thermodynamics of biological processes, including catalysis^55^. The observed modulations of correlations under different conditions suggest allosterically controlled interdomain communications. The quantitative comparisons of best-fit parameters (Table S1) strongly indicate that interdomain dynamics and activity of MMP1 are functionally related and allosteric. Further, a comparison with interdomain dynamics on fibrin (this paper) and collagen^23^ suggest that MMP1 undergoes substrate-dependent and allosterically-controlled dynamics.

### A two-state Poisson process describes MMP1 interdomain dynamics on fibrin

**Figure 4:**
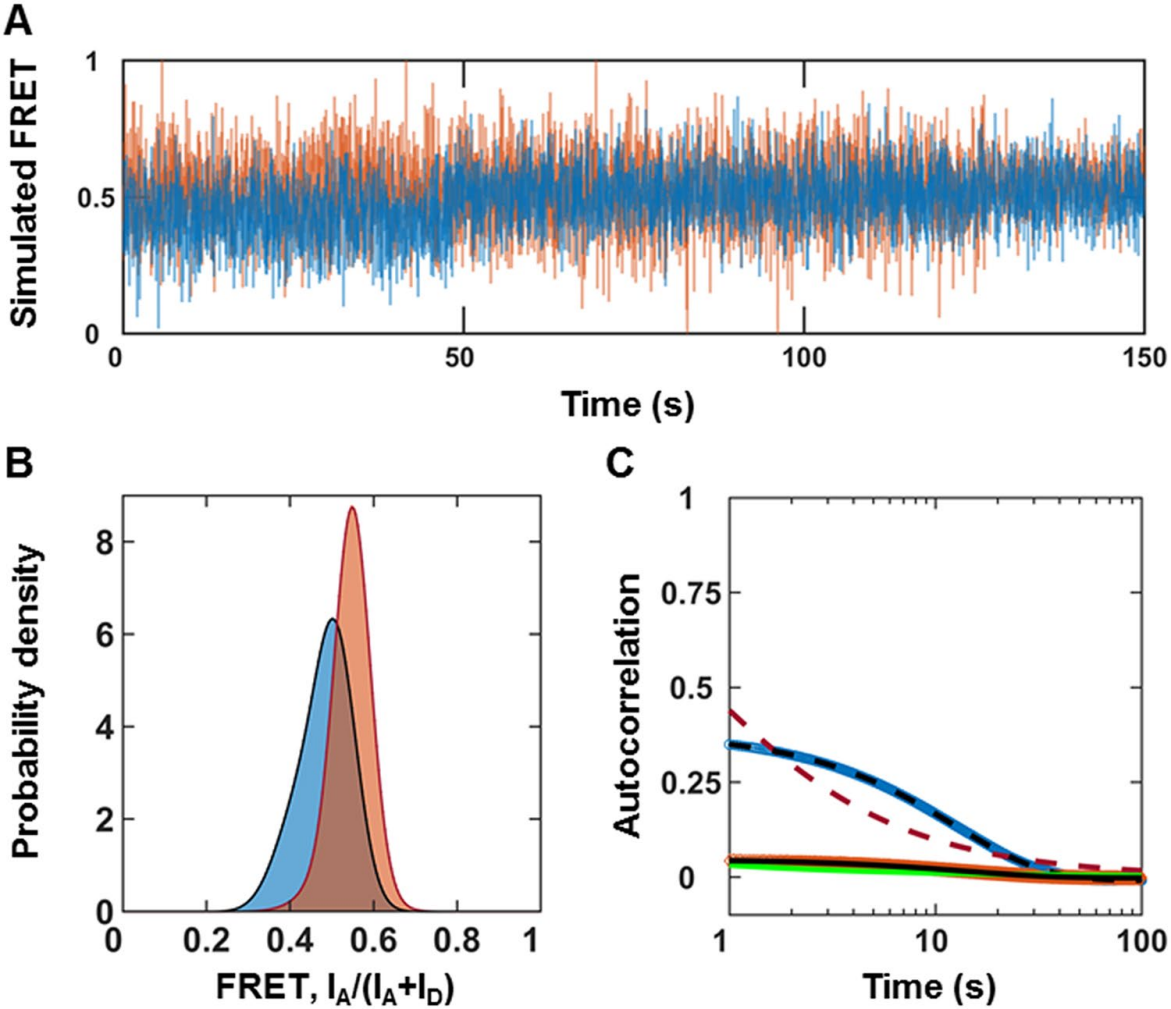
MMP1 interdomain dynamics as a two-state system. (**A**) Examples of simulated smFRET trajectories with noise for active MMP1 (blue) and active site mutant of MMP1 (orange) using experimentally-determined parameters for MMP1 without tetracycline. (**B**) Area-normalized histograms of simulated smFRET values with best fits to a sum of two Gaussians (solid black line). (**C**) Autocorrelations of simulated smFRET trajectories with best fits to exponentials (active: dashed black line; active site mutant: solid black line). As expected, power law did not fit autocorrelations (active: dashed red line; active site mutant: solid green line). k1 + k2 was recovered from exponential fits with and without noise. The error bars are the standard errors of mean for histograms and autocorrelations and are too small to be seen.

While the histograms reveal conformations' distributions, the autocorrelations reveal whether or not conformations at different time points are related. We found that a sum of two Gaussians fits the histograms of smFRET values. Power law fits the autocorrelations for two-state simulations without noise at millisecond timescales and molecular dynamics simulations at picosecond timescales for collagen^23^. As such, we tried to fit both power law and exponential to the autocorrelations for fibrin. The fit equations and best-fit parameters are in the supplementary information. The exponential fits to autocorrelations enable a more straightforward interpretation of the decay rates of correlations if we approximate the conformations of MMP1 as a two-state Poisson process^23^. As a result, we can establish a quantitative connection between the histograms and autocorrelations to calculate the kinetic rates between the two states. To test this, we considered the two centers of Gaussian fits to histograms for MMP1 without ligands (Fig. 3A) as the two states, S1 (low FRET) and S2 (high FRET). We defined the two kinetic rates as k1 (S1 → S2) and k2 (S2 → S1) for interconversion between S1 and S2. We calculated k1/k2 from the ratio of the Gaussian area(S2)/area(S1) and k1 + k2 from the decay rates of autocorrelations for a two-state system. We solved the two equations to calculate k1 and k2 (Table S1C) for both active MMP1 and active site mutant of MMP1. With experimentally-determined S1, S2, k1, k2, and noise (widths of the histograms), we simulated smFRET trajectories for active MMP1 and active site mutant of MMP1. We analyzed simulated data the same way as we did experimental smFRET trajectories (Fig. 4). For comparison, experimentally-determined inputs and recovered parameters from two-state simulations are in Table S2. In summary, we show that a two-state system describes MMP1 interdomain dynamics.

For active MMP1 without ligands, the two states are S1 = 0.42 and S2 = 0.51 on fibrin (Table S1A), similar to the two states S1 = 0.44 and S2 = 0.55 on collagen. However, the correlation decay rate of 0.08 s^−1^ on fibrin (Table S1C) is lower than the rate of 0.13 s^−1^ on collagen. In the presence of MMP9 and tetracycline, the two states and kinetic rates change even though a two-state description still applies for active MMP1 and active site mutant of MMP1 on fibrin (Fig. 3 and Table S1) and collagen. In other words, MMP1 undergoes substrate-dependent interdomain dynamics that follow a two-state Poisson process.

The two-state description also reveals the importance of noise in autocorrelations. Without noise, both power law and exponential fit the autocorrelations of simulated smFRET trajectories (Figure S1). With noise, however, only exponential fits the autocorrelations. Just as noise can convert a Lorentzian line shape into a Gaussian line shape^56^, the noise seems to turn a power law correlation into an exponential one. Note that the exponential fits recover the underlying sum of the simulated kinetic rates with and without noise.

### A larger interdomain distance can accompany larger catalytic pocket opening

**Figure 5:**
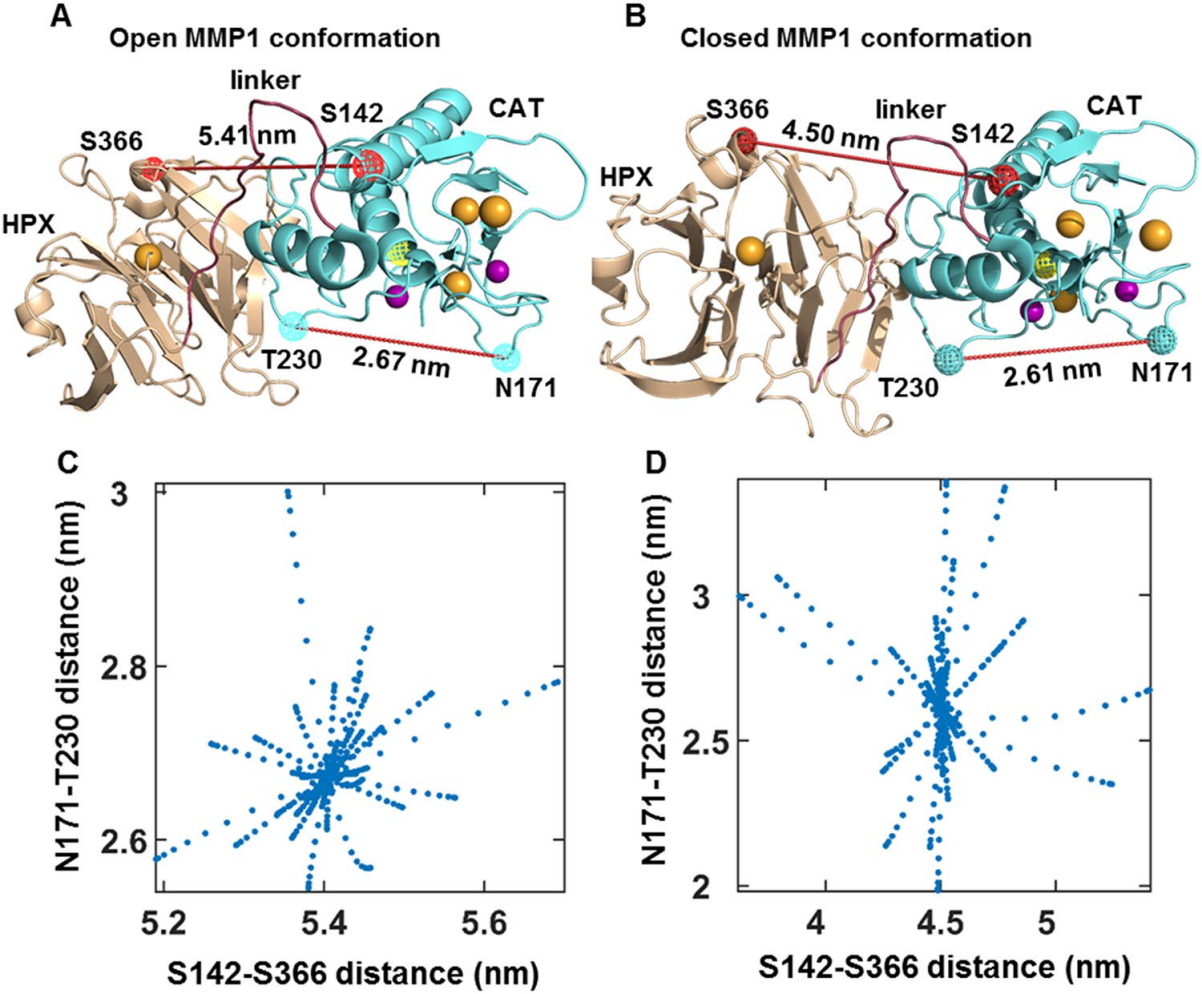
Correlations of MMP1 interdomain distance with catalytic pocket opening when MMP1 is not bound to a substrate. Examples of (**A**) open and (**B**) closed conformations of MMP1. The interdomain distances between S142 and S366 and corresponding catalytic pocketing openings between N171 and T230 have been noted. Spheres and cages represent the van der Waals radii. Yellow cage: E219; Wheat sphere: calcium; Mauve sphere: zinc. (**C**,**D**) are scatter plots of interdomain distance and catalytic pocket opening for the open and closed conformations. Two distances are calculated using ANM simulations (see “Methods”).

The catalytic cleft of MMP1 (~ 0.5 nm) is narrow^17^, and as such, cleavage of substrates such as fibrin monomer (diameter ~ 2–5 nm)^24^ and collagen monomer (diameter ~ 1.5 nm)^57^ needs more opening of the catalytic pocket for easier access to the catalytic cleft. To test this, we used ANM simulations^58^ to calculate normal modes of MMP1 dynamics. ANM models proteins as a system of beads (amino acids) connected by springs (bonds) and calculates the preferred/normal modes of protein motion and corresponding eigenfrequencies. Single molecule experiments (Fig. 3) suggest that both active MMP1 and active site mutant of MMP1 has an open conformation (lower FRET) and a closed conformation (higher FRET). Therefore, we chose two MMP1 conformations (Fig. 5A,B) for ANM simulations (see “Methods” for details). To select the open conformation, we performed coarse-grained simulations of free MMP1 using ANM and decided on the MMP1 conformation with the highest interdomain distance (Fig. 5A). For closed conformation, we selected the conformation in the crystal structure (PDB ID: 1SU3) (Fig. 5B). We considered 20 modes (20 frames for each mode) with the lowest frequencies (slower motion) to calculate the interdomain distance (S142–S366) and catalytic pocket opening (N171-T230). A comparison of Fig. 5C,D shows that MMP1 in the open conformation has an overall catalytic pocket opening (~ 2.7 nm), whereas the closed structure has an opening (~ 2.6 nm). Since a larger catalytic pocket opening enables access to the catalytic cleft, we can infer that larger interdomain distances (lower FRET) correlate with MMP1 activity.

**Figure 6:**
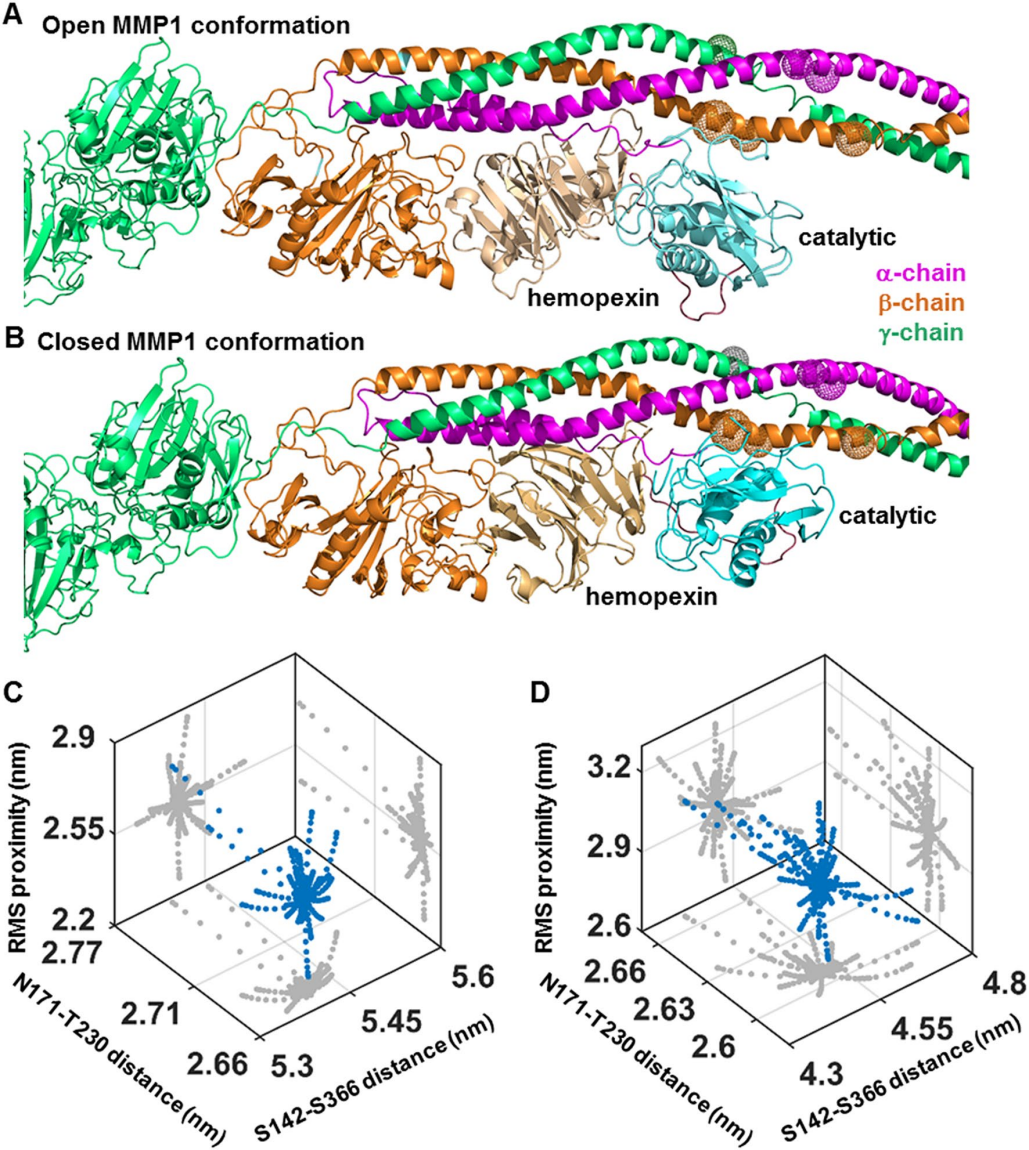
Correlations of MMP1 interdomain distance with catalytic pocket opening when MMP1 is bound to the reconstructed fibrin model combining 3GHG and 1FZC. Examples of (**A**) open and (**B**) closed conformations of MMP1 bound to the reconstructed model of fibrin. Three dimensional scatter plots (blue circle) of interdomain distance (S142–S366), catalytic pocket opening (N171-T230), and rms proximity between the MMP1 catalytic site and the three fibrin chains for (**C**) open and (**D**) closed MMP1 conformations. Two-dimensional projections of the scatter plots are in gray. The open structure shows larger catalytic pocket openings.

To investigate whether or not a larger interdomain distance correlates with a larger catalytic pocket opening when MMP1 is bound to fibrin, we needed to determine the docking poses of MMP1 with fibrin because there is no crystal structure of fibrin-bound MMP1. To this end, open and closed conformations of MMP1 (Fig. 5A,B) were docked to a reconstructed model of fibrin using ClusPro^59^, a protein–protein docking server. The reconstructed model (see “Methods” for detailed procedure) is a combination of the crystal structures of fibrinogen (PDB ID: 3GHG) and fibrin (PDB ID: 1FZC). We sorted the docking poses based on the distance from the α-carbon of E219 at the MMP1 catalytic site to the potential cleaving sites on fibrin. Since the cleavage sites on fibrin for MMP1 are unknown, we considered the cleavage sites for MMP3, MMP7, and MMP14 as substitutes: the α-chain at Asp97-Phe98, and Asn102-Asn103; the β-chain at Asp123-Leu124, Ans137-Val138, and Glu141-Tyr142; and the γ-chain at Thr83-Leu84^54^ (Figure S2). For each docking pose, we calculated the minimum distance possible for the three chains and considered the average of three lengths as the metric for the catalytic domain-fibrin proximity. In addition, we assumed that the hemopexin domain remains bound to the substrate, and thus, we considered the distance from the center of mass (CoM) of the hemopexin domain to any atom of the substrate as the metric for the hemopexin domain-fibrin proximity. We sorted the docking poses according to the two measures of proximity to the MMP1 domains. We selected the docking pose with minimum scores for ANM simulations' two criteria (Fig. 6A,B). We considered 20 modes (20 frames for each mode) with the lowest frequencies (slower motion) to calculate the interdomain distance (S142–S366), catalytic pocket opening (N171-T230), and rms proximity between the MMP1 catalytic site and the three fibrin chains (Fig. 6C,D). The interdomain distance reduces after binding fibrin for both the open and closed conformations of MMP1. As is the case of free MMP1 (Fig. 5C,D), fibrin-bound MMP1 also showed that larger interdomain distance for the open conformation accompanies larger catalytic pocket openings (Fig. 6C,D). In contrast, fibrinogen-bound MMP1 shows a larger catalytic pocket opening for the closed conformation (Figure S3). The importance of open conformations on fibrin and closed conformations on fibrinogen is an experimentally testable insight from ANM simulations. We could test with fibrinogen attached to a quartz slide for TIRF imaging at the single molecule level. Note that the overall charge is negative for both fibrinogen and a clean quartz slide, making it challenging to attach fibrinogen on a quartz slide. Nevertheless, histograms of smFRET values would be peaked at a higher FRET value on fibrinogen than fibrin. One could also try to crystalize MMP1 with fibrinogen and fibrin and check the conformations. Nevertheless, both fibrinogen- and fibrin-bound MMP1 show closer proximity to the three chains for the open conformation. The proximity suggests that a larger interdomain distance is relevant for MMP1 activity.

### The proximity of MMP1 to the three chains correlates with the sequence of degradation

**Figure 7:**
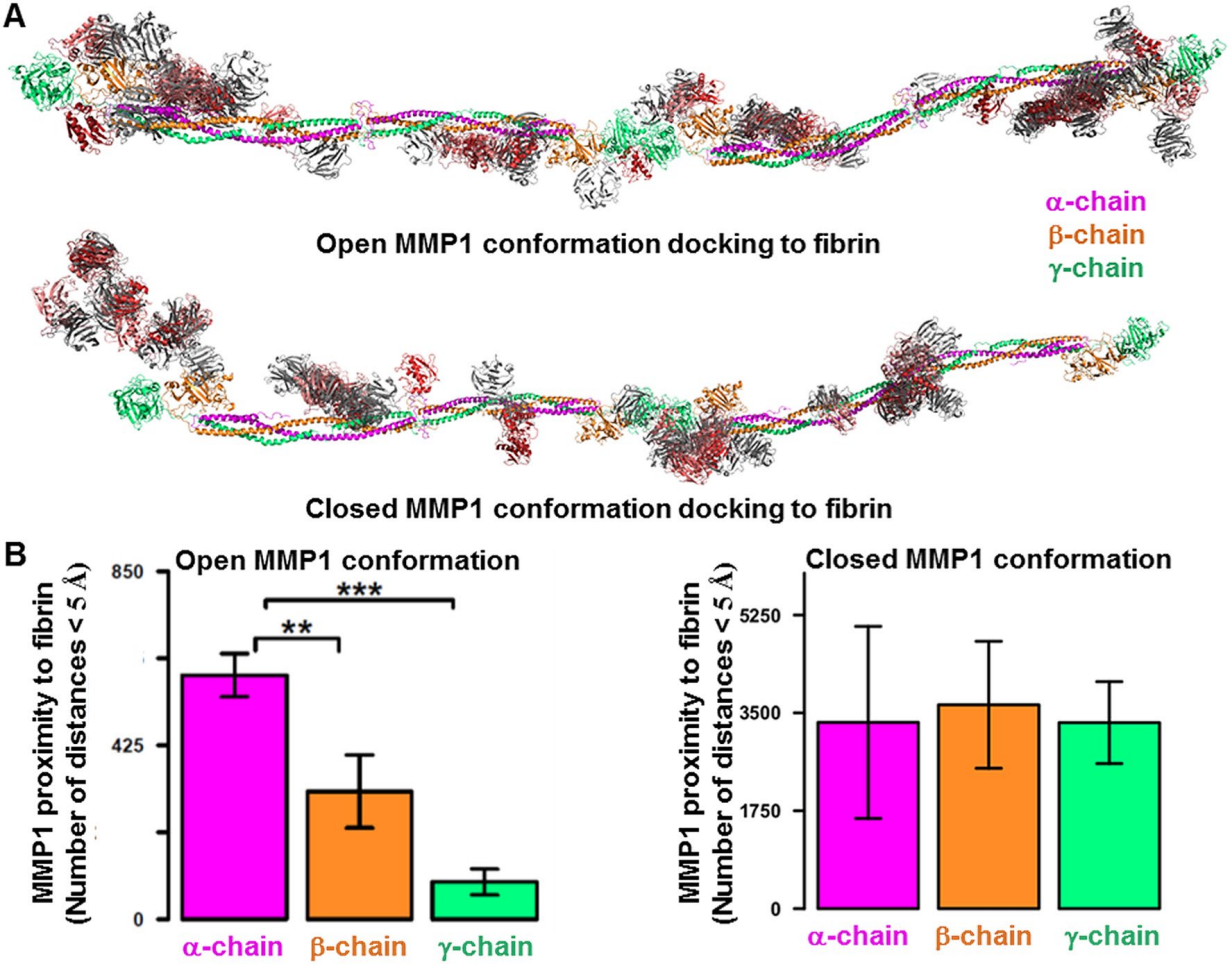
Proximity of MMP1 to the chains of reconstructed fibrin model. (**A**) The clustered 30 docking poses obtained from ClusPro using fibrin as the ligand and MMP1 as the receptor. The catalytic domain of MMP1 is red, whereas the hemopexin domain is gray. (**B**) We measured the distance between all possible pairs of atoms between the fibrin chains and MMP1 for 30 docking poses obtained from ClusPro. We counted any distance less than 5 Å and plotted the total count distributions for each chain for the open and closed conformations. p-value < 0.05:*; p-value < 0.01:**; p-value < 0.001:***

Since MMP1 needs to come in proximity before cleavage, we performed molecular docking of MMP1 with fibrin. Figure 7A shows MMP1 molecular docking poses superimposed on the reconstructed model of fibrin. MMP1 binds to fibrin at specific places in both open and closed conformations. We calculated the distance between every atom in MMP1 and the three fibrin chains to gain further insights. We counted the number of distances below 0.5 nm for the top 30 docking poses and calculated the mean and standard deviation (Fig. 7B). On average, MMP1 in open conformation showed the closest proximity to the α-chain (Fig. 7B, left panel). We quantified the statistical significance by p-value (Fig. 7B, left panel). In contrast, the closed conformation of MMP1 did not show any significant difference in proximity to the three chains (Fig. 7B, right panel).

To test whether or not the sequence of MMP1 proximity to the three chains has any implication in fibrinolytic activity, we performed the time-dependent fibrinogen degradation. Figure S4B shows that the overall catalytic activity is fastest on the α-chain and slowest on the γ-chain. Fibrinogen, the basic building block of fibrin, has three pairs of amino acid chains: (1) the α-chain at ~ 63 kDa, (2) the β-chain at ~ 56 kDa, and (3) the γ-chain at ~ 47 kDa. SDS PAGE of fibrinogen control approximately confirms the three chains' molecular weights (Figure S4A, lane 2 from left). These three chains are connected by a dimeric disulfide knot (DSK) at the N-termini and many other disulfide bonds along the length of fibrinogen^60,61^. Treatment of 1 mg/mL of fibrinogen with 0.1 mg/mL of active MMP1 at 37 °C for 24 h resulted in cleavage of all three chains and a final prominent fragment at ~ 40 kDa (Figure S4A, lane 3 from left). Cleavage due to MMP1 leads to complete disappearance of the α-chain and partial disappearance of the β-chain within 2 h; however, the γ-chain took nearly 6 h to be degraded (Figure S4B). After 8 h, a prominent band at ~ 40 kDa remained. In other words, the computationally determined sequence of degradation in the open conformation agrees with the experimentally observed sequence (Figure S4B). The agreement between the computational and experimental sequence of degradation further confirms that the open conformations observed in smFRET experiments are functionally relevant.

We measured the activity of MMPs on fibrin without crosslinks (Figure S4C), which showed bands at positions lower than the bands for fibrinogen (Figure S4A) due to cleavage by thrombin. Also, MMP1 appears to degrade the β-chain in fibrin slower than the β-chain in fibrinogen. One plausible explanation is that MMP1 has reduced access to the β-chain in fibrin due to polymerization.

We also imaged fibrin without crosslinks after treatment with MMP1 using SEM (Figure S4D), which shows increased porosity of fibrin for active MMP1 consistent with the degradation. We used fibrin without crosslinks because it is easier to form a thin layer of fibrin on a quartz slide for smFRET measurements, and the crystal structure without crosslinks (PDB ID: 1FZC) is available. However, fibrin with crosslinks is more physiologically relevant (see “Methods” for making fibrin with crosslinks). As such, we quantified the degradation rate of fibrin with crosslinks using a weight-based assay (Figure S5A). The rate of crosslinked fibrin degradation by MMP1 is ~ 0.23 mg/h/μg at 37 °C. Figure S5B shows the effects of tetracycline and MMP9 on MMP1 activity. Complementary to the single molecule measurements, the weight-based assay supports that tetracycline inhibits and MMP9 enhances MMP1 activity on crosslinked fibrin. The rates of degradation by MMP9 alone and MMP1 with MMP9 are ~ 15 mg/h/μg and ~ 20 mg/h/μg, respectively. The combined activity of MMP1 and MMP9 shows a step, suggesting cooperativity between the two enzymes. We also performed gel electrophoresis of degradation product (Figure S6A), imaged the reaction volume to show the degradation within 5 h (Figure S6B), and imaged the surface morphology using SEM (Figure S6C).

### Unlike collagen, MMP1 domains do not communicate via fibrin

**Figure 8:**
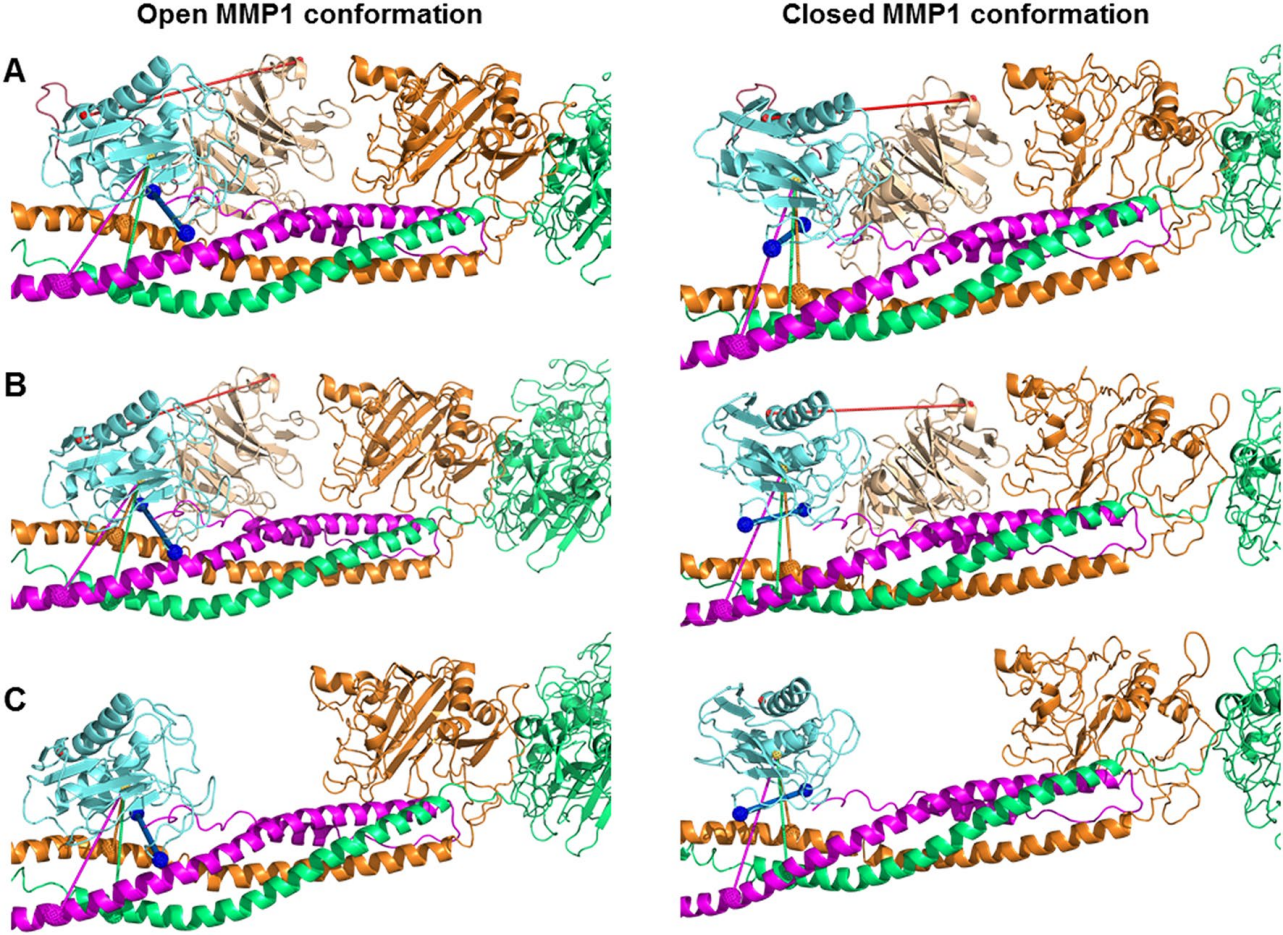
Effects of the linker and reconstructed fibrin on the catalytic pocket opening. The catalytic pocket openings on fibrin represented by the blue line connecting N171 and T230 for (**A**) the full-length MMP1 (open conformation: 2.68 ± 0.01 nm; closed conformation: 2.61 ± 0.01 nm); (**B**) the two MMP1 domains without the linker (open conformation: 2.68 ± 0.01 nm; closed conformation: 2.61 ± 0.01 nm); and (**C**) the catalytic domain alone (open conformation: 2.68 ± 0.01 nm; closed conformation: 2.68 ± 0.01 nm). The error bars represent the standard deviation of 60 measurements of the catalytic pocket opening obtained from 20 frames each for the three slowest normal modes.

Previously, we performed ANM simulations of MMP1 interacting with collagen using the crystal structure of MMP1 bound to a collagen model (4AUO)^23^. We reported that the catalytic and hemopexin domains could communicate via collagen even if the physical linker is removed^23^, which explained the experimental observation that a mixture of the two domains purified separately could degrade triple-helical collagen^18^. We wanted to investigate whether or not the two domains communicate via fibrin. We created three forms of MMP1 using the crystal structure (PDB ID: 1SU3): (1) full-length MMP1, (2) MMP1 with the linker domain removed, and (3) MMP1 with the linker and hemopexin domain removed. We docked both the open and closed conformations with the reconstructed fibrin using ClusPro and chose the binding pose shown in Fig. 8. Three forms of MMP1 interacting with fibrin resulted in similar mean and standard deviation of the catalytic pocket opening (Fig. 8). In contrast, the catalytic pocket opening on collagen changed even without the physical linker^23^. In other words, fibrin does not lead to more catalytic pocket opening, whereas collagen can lead to a more catalytic pocket opening. Using single molecule tracking of labeled MMP1 on type-1 collagen fibrils, we also showed that fibrils play a role in MMP1 activity due to vulnerable sites on fibrils created during fibril assembly^62^.

Both fibrin and collagen are extracellular and triple-helical but have microstructural and mechanical differences^63^. Fibrin monomers are 45 nm long and have three right-handed chains forming a left-handed structure between two globular regions^24^. In contrast, collagen monomers are 300 nm long and have three left-handed chains forming a right-handed structure^25^. The difference in handedness between fibrin and collagen may lead to in-phase and out-of-phase motion of MMP1 domains. However, to understand why fibrin and collagen interact differently, we need further studies since fibrin and collagen have different sequences and structures despite the triple-helical similarities.

In summary, we have shown that the interdomain dynamics of MMP1 on fibrin correlate with the fibrinolytic activity of MMP1. We measured smFRET between two fluorophores attached to the catalytic and hemopexin domains of MMP1. A comparison of distributions of smFRET values between active MMP1 and active site mutant of MMP1 suggests that MMP1 needs to have the two domains well-separated for function. To investigate the roles of open conformations, we measured smFRET in the presence of MMP9 and tetracycline. MMP9 is an enhancer, whereas tetracycline inhibits MMP1 activity on collagen and a synthetic substrate. In the presence of tetracycline, the interdomain distance on fibrin becomes smaller consistent with the trend on collagen. MMP9 causes the interdomain distance of MMP1 to be shorter on fibrin, which is opposite to the observation on collagen. One possible reason is that MMP9 by itself can degrade fibrin but cannot degrade triple-helical collagen. As a result, fibrin degradation by MMP1 in the presence of MMP9 is more complicated. A two-state Poisson process quantitatively describes the interdomain dynamics. We fitted the histograms of smFRET values to a sum of two Gaussians. The best-fit parameters for the centers are the two states used for simulations. The ratio of kinetic rates equals the ratio of areas of the two Gaussians fitted to a histogram. Additionally, the sum of kinetic rates equals the decay rate of autocorrelations of smFRET values. We simulated smFRET trajectories with experimentally determined parameters and noise levels. We recovered the underlying parameters to validate our analyses. The presence of noise in the signal appears to convert a power law autocorrelations into an exponential one.

We performed molecular docking of the open and closed conformations of MMP1 with fibrin. In the closed conformation, MMP1 approaches the three chains equally. However, in the open conformation, MMP1 approaches the α-chain first and the γ-chain last, which we confirmed using SDS PAGE of fibrinogen degraded by MMP1. As such, the open conformation appears to be more functionally relevant. One or both domains of MMP1 binds to fibrin at many places along the length of fibrin. However, the primary location on fibrin is near the globular regions where both the domains bind. We, therefore, selected the docking pose near the globular part for ANM simulations. ANM simulations of MMP1-fibrin interactions revealed that the open conformation with well-separated domains has a larger catalytic pocket opening. A larger opening enables the fibrin chains to approach closer to the MMP1 active site. In the closed conformation, the fibrin chains get closer to the catalytic site only when the catalytic domain is alone. We performed limited digestion of fibrin by MMP1 with and without crosslinks and observed increased porosity due to degradation. Also, MMP1 degrades and dissolves crosslinked fibrin within 5 h, suggesting a role of MMP1 in the fibrinolytic pathway. We quantified the degradation using a weight-based assay because interactions of water-soluble MMP1 with water-insoluble fibrin are challenging to quantify using solution-based biochemical assays.

We discussed the results of the MMP1-fibrin system in light of our previous studies on the MMP1-collagen system^23^. For both fibrin and collagen, the open conformation of MMP1 is functionally relevant and responds to tetracycline similarly. The open conformation enables larger catalytic pocket opening and closer proximity to the three strands of fibrin and collagen. A two-state Poisson process describes MMP1 dynamics on both fibrin and collagen. However, MMP1 dynamics are opposite to each other on fibrin and collagen in the presence of MMP9. Note that MMP9 can degrade fibrin but cannot degrade triple-helical collagen and, as such, can influence MMP1 dynamics differently. Collagen plays an active role in mediating allosteric communications between the two domains and opening the catalytic pocket. In contrast, although fibrin also plays an active role in mediating allostery, the allosteric communications of MMP1 on fibrin do not increase the catalytic pocket's opening. One possible explanation for this difference could be the difference in strains in collagen and fibrin backbones. We reported that the backbone strain in collagen could change the interdomain dynamics and catalytic pocket opening of MMP1^23^. Further studies are needed with different backbone strains to investigate the mechanisms of MMP1 dynamics on fibrin and collagen, which may enable allosteric control of MMP1 activity in a substrate-dependent manner for exploring MMPs as drug targets.

## Methods

### Purification and labeling of MMP1

Full-length recombinant MMPs were expressed in *E. coli* and activated using 0.1 mg/mL trypsin. Activated MMP1 and trypsin create a chain reaction of further MMP1 activation and broad-spectrum proteolytic cleavage of *E. coli* proteins. Molecular weights of activated MMP1 and trypsin are ~ 43 kDa and ~ 23 kDa, respectively. We used a 30 kDa cut-off Amicon filter to centrifuge and purify MMP1 in the activated form. For further details, see the previous publication on protease-based purification method^14^. We labeled purified MMP1 with AlexaFluor555 (ThermoFisher Scientific, Cat# A20346, donor dye) and AlexaFluor647 (ThermoFisher Scientific, Cat# A20347, acceptor dye) in a ratio of ~ 1:1 using maleimide chemistry. 1 mL of MMP1 at 1 mg/mL concentration was incubated with 20 µL of 1 mg/mL AlexaFluor555 and AlexaFluor647 for 1 h in a 5 mL glass vial in a nitrogen environment to avoid oxidation of the dyes. After incubation, we used a 30 kDa cut-off Amicon filter to remove free dyes from the solution. We compared the specific activities of labeled and unlabeled MMP1 on the synthetic substrate, MCA-Lys-Pro-Leu-Gly-Leu-DPA-Ala-Arg-NH2, (R&D Systems, Cat# ES010) as described before^14^. Labeling MMP1 does not affect its specific activity^23^.

### Single-molecule measurements

We prepared 200 µL of reaction volume in 10 mM Phosphate Buffer Saline (PBS) (Sigma, Cat# P3813, pH 7.4) by mixing 10 units of thrombin (Cayman chemical, Cat# 13188) and 50 μg of fibrinogen (Cayman chemical, Cat# 16088). A thin layer of fibrin was created on a quartz slide similar to the blood smear protocol used in diagnostics^64^ and incubated at 37 °C for 18 h without shaking. We made a flow cell of thickness 120 μm using a piece of double-sided adhesive tape, a clean quartz slide, and a glass coverslip. The quartz slides had two holes to connect the input and output tubes for exchanging buffers and solutions. We created an evanescent wave at the slide's interface in a Total Internal Reflection Fluorescence (TIRF) Microscope, as described before^39,40,65,66^. We incubated 50 µL of 0.1 mg/mL labeled MMP1 with (1) 50 µL of protein buffer (50 mM Tris, 100 mM NaCl, pH 8.0), (2) 50 µL of 1 mg/mL MMP9, and (3) 50 µL of 0.1 mg/mL tetracycline for 30 min at 22 °C. The labeled MMP1 was serially diluted to prepare a working concentration of ~ 100 pM and flowed into the flow cell. Alexa555 dyes (donor dyes) attached to MMP1 was excited using a 532 nm laser and imaged using the TIRF microscope at 22 °C at 100 ms time resolution. We imaged Alexa647 and Alexa555 emissions using an EMCCD camera (Andor iXon).

We superimposed the two emission channels using a pairwise stitching plugin of ImageJ, and the intensities of the two dyes were extracted and analyzed using Matlab. We calculated more than 300,000 FRET values from the intensities of the two dyes for each condition. After creating the histograms of FRET values, we calculated the total area under the curve. We divided each bin count by the total area to get the area-normalized histograms, which give the probability densities. We calculated the error bars at each bin by calculating the square roots of bin counts and divided by the total area to normalize the error bars. The total area under each area-normalized histogram is 1, consistent with the requirement that the probability densities integrate to 1 over all possible values of FRET. As a control to confirm fibrin-specific interaction of MMP1, we made a flow cell without fibrin and flowed labeled MMP1. In a field of view with dimensions 80 μm × 80 μm, we observed no more than 2–5 spots when the slides were clean. In contrast, there were many spots when the flow cell had fibrin. The presence of persistent bright spots lasting several minutes suggests that MMP1 binds to fibrin with a strong affinity. Otherwise, labeled MMP1 would quickly diffuse out in solution within a few μs and appear as a single-step reduction of intensity. Photobleaching of dyes also leads to a single-step decrease in emission. However, photobleaching of Alexa dyes happens in 2–3 min on average for the laser excitation used in the experiment (~ 1 mW of 532 nm laser, focused using a 10 cm plano-convex lens)^65,66^.

### Reconstruction of the extended fibrin molecule

We superimposed the crystal structure of fibrinogen (PDB ID: 3GHG)^67^ with the crystal structure of D-D fibrin fragment (PDB ID: 1FZC)^68^ using the Combinatorial Extension (CE) algorithm^69^ implemented in PyMOL^70^ to obtain the reconstructed model of fibrin. The fibrinogen asymmetric unit presents two replicas (either the chains A–F + M–P or G–L + Q–T). Each replica has two ends: chains A–C and chains D–F in one and chains G–I and J–L in the other. Each of such fibrinogen ends could be superimposed to any of the ends of the fibrin crystal (either the chains A–C or chains D–F), yielding a total of 16 different possibilities to reconstruct the fibrin molecule. For each superimposed assembly, we superimposed a new fibrin crystal to the fibrinogen biological assembly not used for the initial superimposition. Thus, we superimposed two extra fibrin crystals (Fa and Fb, one per superimposed fibrinogen biological assembly) on each of the 16 different reconstruction attempts. A reconstruction compatible with the original fibrinogen packing would show no differences in Fa and Fb's positioning. Thus, to select the best superimposition combination to reconstruct the fibrin molecule, the one that minimized the RMSD in between Fa and Fb was selected.

### Selection of closed and open conformations of MMP1

The activation peptide (residues 32 to 98) was removed from the first biological assembly (chain A) of the crystal structure of MMP1 (PDB ID: 1SU3), leaving only the catalytic (residue numbers 107 to 260), linker (261–277), and hemopexin (278–466) domains of the enzyme, which we selected as the closed conformation. To obtain the open conformation, we submitted the closed conformation coordinates to the ANM web server 2.1^58^ using default parameters. We extracted the coordinates of the three-dimensional fluctuations for 20 frames of the 20 slowest modes. For each frame on each model, we calculated distances using PyMOL. We used the distance between S142 and S366 to analyze the fluctuations between the catalytic and hemopexin domains. We selected the frame that maximized this distance (second mode, second frame) as the MMP1 open conformation.

### Molecular docking of MMP1 with fibrin

The closed and open conformations of MMP1 were docked individually with the reconstructed fibrin molecule using the default parameters of ClusPro 2.0 web server^59^. We used the reconstructed fibrin as the receptor and the MMP1 conformations as the ligands. We considered the centroid structure of each of the resulting clusters (maximum 30) as the representative docking solution for a cluster. We obtained the corresponding weighted scores for the best coefficient for each binding pose. We adjusted different poses to the same Cartesian space by aligning the reconstructed fibrin from each pose to a template one using PyMOL. To analyze the accessibility of MMP1 to the three chains, we counted the number of times an atom in MMP1 was within 5 Angstrom from an atom in the chains.

### Preparation of fibrin without and with crosslinks

We prepared fibrin without crosslinks by adding 10 units of thrombin (Cayman chemical, Cat# 13188) to 50 μg of fibrinogen (Cayman chemical, Cat# 16088) in 10 mM PBS to a total reaction volume of 200 μL and incubating at 37 °C for 18 h with 250 rpm shaking inside an incubator (Thermo Fisher Scientific, MaxQ600). Note that the molar concentration of thrombin can be estimated by considering the specific activity of thrombin to be ~ 3000 units/mg. Preparation of crosslinked fibrin involved two additional reagents: factor XIII (Abcam, Cat # ab62427) and CaCl_2_. Thrombin activates factor XIII to factor XIIIa, which in the presence of Ca^2+^ induces crosslinks in fibrin. We added 10 units of thrombin to 2.5 µg of factor XIII in a 0.5 mL PCR tube, diluted with 10 mM PBS to a total reaction volume of 100 μL, and incubated at 37 °C for 10 min. After incubation, we added 50 μg of fibrinogen and 10 µL of 5 mM CaCl_2_, diluted with PBS buffer to a total reaction volume of 200 µL, and incubated at 37 °C for 15 min to obtain gel-like crosslinked fibrin. Note that fibrin without crosslinks requires a longer incubation time and shaking, but fibrin with crosslinks forms within 15 min without shaking. For every reaction, we put a part of the sample on a sample mount before drying and imaging the surface morphology of fibrin using a Phenom Pro Scanning Electron microscope.

### Sodium dodecyl sulphate–polyacrylamide gel electrophoresis (SDS PAGE)

The degradation profile after fibrinolysis was analyzed using SDS-PAGE. We mixed SDS PAGE loading buffer containing BME (Biorad, Cat# 610737) with each sample in a 1:1 (v/v) ratio and incubated at 95 °C for 10 min. We loaded the samples onto the wells of 8% Tris.glycine gels. We ran electrophoresis in Tris.glycine buffer (Biorad, Cat# 1610732) for 30 min at 50 V, followed by 1 h at 120 V. We used the precision plus protein kaleidoscope pre-stained protein standards (Biorad, Cat# 1610375) as molecular weight markers. The SDS-PAGE gel was stained with Coomassie Brilliant Blue R-250 (Biorad, Cat# 161-0436) and imaged using a digital camera after destaining.

For fibrin without crosslinks, we prepared fibrin samples in 1.5 mL Eppendorf warmed to 37 °C by adding 40 µL of 25 mg/mL fibrinogen (Cayman chemical, Cat# 16088), 20 µL of 1000 U/mL thrombin (Cayman chemical, Cat# 13188), and 40 µL of 0.01 M PBS. The mixture became cloudy and visible threads formed immediately after the addition of thrombin. Samples were vortexed for 1 s and then centrifuged quickly for 3 s at 10,000 rpm to collect all the samples to the tube's bottom. We incubated samples at 37 °C without shaking for 15 min. Samples were then treated for 18 h either with 100 µL of 2.5% Triton buffer (2.5% Triton X-100 dissolved in 50 mM Tris, 100 mM NaCl, pH 9.0), 100 µL of 0.12 mg/mL active MMP1, 100 µL of 0.12 mg/mL inactive MMP1, and 100 µL of 0.14 mg/mL MMP9. The next morning, all four samples were prepared for SDS PAGE by 1:1 dilution in sample buffer with β-mercaptoethanol (Sigma-Aldrich, Cat# M3148) prepared from 2X Laemmli buffer (Biorad, Cat# 161-0737). We boiled the samples for 10 min after adding the dye and loaded 30 μL of each sample into the wells of an 8% polyacrylamide gel. The gel was run in 1X SDS running buffer at 70 V. After 30 min, we increased the voltage to 120 V, and gels were allowed to run for an additional 1 h. Gels were removed from the running tank and stained in 100 mL of stain prepared using 30% ethanol, 10% acetic acid, and 0.5% Coomassie Brilliant Blue R-250 (Biorad, Cat# 161-0436). The gel was stained for 2 h and washed twice in DI water to remove excess dye, followed by destaining in 200 mL of 30% ethanol and 10% acetic acid for 24 h. We imaged the gel using a digital camera.

## Supplementary information

Supplementary Information

## Data Availability

The datasets generated during and/or analyzed during the current study are available from the corresponding author on reasonable request.
