# Supplementary material for: Activity-dependent interdomain dynamics of matrix metalloprotease-1 on fibrin
Source: Sci Rep. 2020 Nov 26;10:20615. doi: 10.1038/s41598-020-77699-3 (PMC7692495; doi:10.1038/s41598-020-77699-3)
Supplement: Supplementary file 1 — Supplementary Information [file 41598_2020_77699_MOESM1_ESM.pdf]

Supplementary Information

Activity-dependent interdomain dynamics of matrix metalloprotease-1 on fibrin

Lokender Kumar[1,#], Joan Planas-Iglesias[2,3#], Chase Harms[1], Sumaer Kamboj[1], Derek Wright[1], Judith Klein-Seetharaman[2,4], and Susanta K. Sarkar [1, *]

[1]Department of Physics, Colorado School of Mines, 1500 Illinois Street, CO 80401, USA
[2]Warwick Medical School, University of Warwick, Coventry CV4 7AL, UK
[3]Loschmidt Laboratories, Department of Experimental Biology, Faculty of Science, Masaryk University, Kamenice 5/A13, 625 00 Brno, Czech Republic
[4]Department of Chemistry, Colorado School of Mines, 1500 Illinois Street, CO 80401, USA

[#]These authors contributed equally

Corresponding author:

[*]ssarkar@mines.edu

We fitted a sum of two Gaussians to the experimental histograms using the following equation:

$$y = a_1 \times e^{-\frac{(x-b_1)^2}{c_1^2}} + a_2 \times e^{-\frac{(x-b_2)^2}{c_2^2}}$$

where a, b, and c are amplitude, center, and width of the Gaussian. The parameters b1 and b2 are the two states, S1 (low FRET) and S2 (high FRET).

Table S1. Best-fit parameters for experimental histograms and autocorrelations. (**A**) A sum of two Gaussians fits the experimental histograms in **Figure 3**. (**B**) An exponential distribution fits the experimental autocorrelations in **Figure 3.** Power law distribution does not fit the experimental autocorrelations. (**C**) Kinetic rates of interconversion between the two states, S1 and S2, from the histograms and autocorrelations. S1 and S2 in Table S1B are the centers of Gaussian fits, and e in Table S1B is the decay rate of exponential fit. The error bars represent the standard errors of mean.

A Gaussian fit parameters for experimental histograms

	MMP1 without ligands		MMP1 with MMP9		MMP1 with tetracycline	
	Active	Inactive	Active	Inactive	Active	Inactive
**a1**	1.99±0.13	0.90±0.17	2.14±0.06	1.18±3.21	4.38±0.05	0.60±0.79
**b1/S1**	0.42±0.01	0.52±0.01	0.46±0.01	0.46±0.18	0.61±0.01	0.49±0.13
**c1**	0.08±0.01	0.10±0.01	0.11±0.01	0.11±0.06	0.10±0.01	0.11±0.06
**a2**	5.52±0.15	8.12±0.17	5.58±0.07	4.61±4.12	2.10±0.12	5.6±1.21
**b2/S2**	0.51±0.01	0.55±0.01	0.51±0.01	0.53±0.01	0.70±0.01	0.58±0.01
**c2**	0.07±0.01	0.06±0.01	0.06±0.01	0.09±0.01	0.06±0.01	0.09±0.01

B Exponential fit parameters for correlations

	MMP1 without ligands		MMP1 with MMP9		MMP1 with tetracycline	
	Active	Inactive	Active	Inactive	Active	Inactive
**d**	0.37±0.01	0.15±0.01	0.21±0.01	0.45±0.01	0.62±0.01	0.36±0.01
**e**	0.08±0.01	0.07±0.01	0.09±0.01	0.05±0.01	0.05±0.01	0.06±0.01
**f**	-0.03±0.01	-0.01±0.01	-0.01±0.01	-0.03±0.01	-0.05±0.01	-0.004±0.001

C Kinetic rates calculated from histograms and correlations

	MMP1 without ligands		MMP1 with MMP9		MMP1 with tetracycline	
	Active	Inactive	Active	Inactive	Active	Inactive
**k1 (s^{-1})**	0.0567	0.0591	0.0528	0.0381	0.0112	0.0531
**k2 (s^{-1})**	0.0233	0.0109	0.0372	0.0119	0.0388	0.0069

We subtracted the average FRET value from each FRET trajectory and used the following equation to calculate autocorrelations:

$$C_\tau = \frac{1}{N-\tau} \sum_{t=0}^{N-\tau} \left\{ I(t) - \frac{1}{N-\tau} \sum_{t'=0}^{N-\tau} I(t') \right\} \times \left\{ I(t+\tau) - \frac{1}{N-\tau} \sum_{t'=\tau}^{N} I(t') \right\}$$

where C_τ is the autocorrelation at lag number τ, N is the number of points in a FRET trajectory, and $I(t)$ is the FRET value at t.

We normalized autocorrelations by dividing autocorrelations at each lag by $C_{\tau=0}$. We fitted autocorrelations between $\tau = 1$ and $\tau = 1000$ to both power law and exponential distributions. For power law, we used a form of Pareto distribution[1] that satisfies the boundary conditions of our calculated autocorrelations, i.e., $C_{\tau=0} = 1$ at $t = 0$ and $C_{\tau=\infty} = 0$ at $t = \infty$. We fitted the following equations of power law and exponential functions:

$$C_{\tau} = \left(a \times \tau + 1\right)^{-b}$$

$$C_{\tau} = d \times \exp^{-e \times \tau} + f$$

Best-fit parameters for two-state simulations. We simulated smFRET trajectories assuming that MMP1 undergoes interconversion between two states, S1 and S2, which are Gaussian's centers that fit the histograms. We considered active MMP1 and active site mutant of MMP1 without ligands (**Figure 3A**). We simulated 350 smFRET trajectories, each 1000 s long, with the input parameters in **Table S2**. We analyzed the simulated and experimental trajectories similarly. The recovered parameters (**Table S2**, right side) agree well with the input parameters.

Table S2. MMP1 interdomain dynamics as a two-state Poisson process. Analysis of FRET trajectories for MMP1 without ligands recovered the centers and decay rates used as inputs. The agreement between the input and retrieved values suggests that a two-state Poisson process describes the MMP1 dynamics.

Input parameters

Active MMP1
S1 = 0.42, S2 = 0.51
k1 = 0.06 s^{-1}, k2 = 0.02 s^{-1}
k1 + k2 = 0.08 s^{-1}
σ1 = 0.08, σ2 = 0.07

Inactive MMP1
S1 = 0.52, S2 = 0.55
k1 = 0.06 s^{-1}, k2 = 0.01 s^{-1}
k1 + k2 = 0.07 s^{-1}
σ1 = 0.10, σ2 = 0.06

Recovered parameters

Gaussian fit parameters for simulated histograms

	Without noise		With noise	
	Active	Inactive	Active	Inactive
a1			2.06±0.01	0.91±0.02
b1/S1	0.42	0.52	0.42±0.01	0.52±0.01
c1			0.08±0.01	0.10±0.01
a2			5.70±0.02	7.91±0.02
b2/S2	0.51	0.55	0.51±0.01	0.55±0.01
c2			0.07±0.01	0.06±0.01

Power law and exponential fit parameters for correlations

	Without noise		With noise	
	Active	Inactive	Active	Inactive
a	0.001±0.001	0.001±0.001	NA	NA
b	1882±1354	2080±1723	NA	NA

	Without noise		With noise	
	Active	Inactive	Active	Inactive
d	1.03±0.01	1.03±0.01	0.39±0.01	0.05±0.01
e	0.08±0.01	0.07±0.01	0.08±0.01	0.07±0.01
f	-0.03±0.01	-0.04±0.01	-0.01±0.01	-0.01±0.01

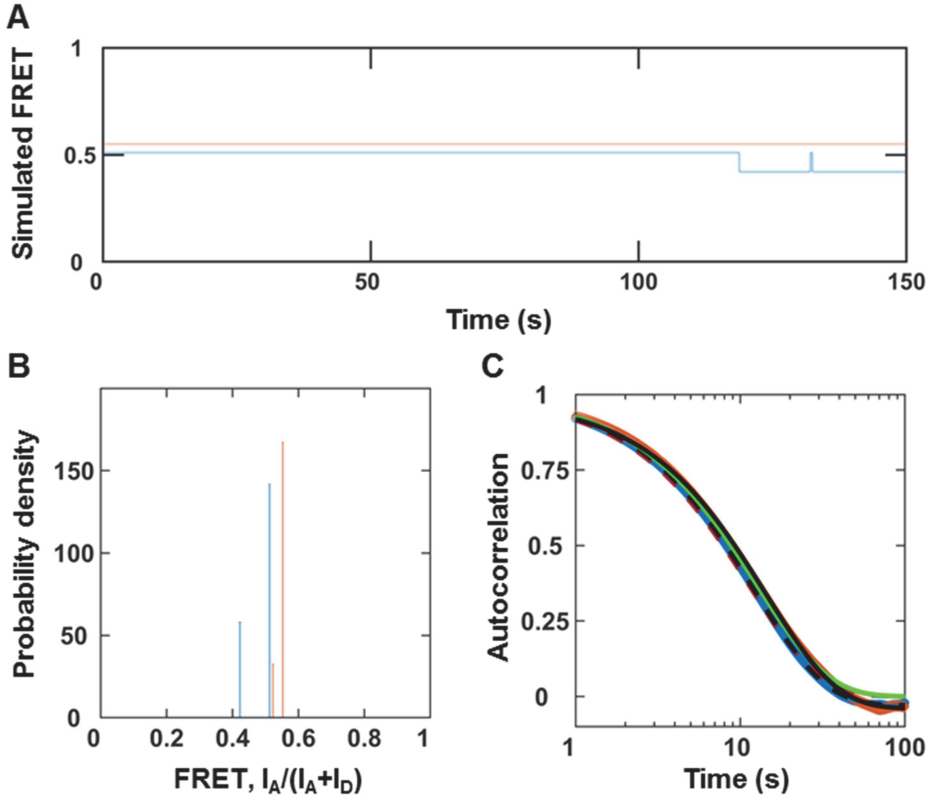

Figure S1. MMP1 interdomain dynamics as a Poisson process without noise. (**A**) An example of simulated two-state FRET trajectory without noise for active MMP1 (blue) and active site mutant of MMP1 (orange). (**B**) Histograms of the recovered FRET values with bin size=0.005. (**C**) Autocorrelations of simulated trajectories recover the sum, k1+k2, from exponential fits (active MMP1: dashed black line; active site mutant of MMP1: solid black line). Note that power law does not fit the autocorrelations with noise (**Fig. 4**). However, power law fits the autocorrelations without noise (active MMP1: dashed red line; active site mutant of MMP1: solid green line). The error bars are the sems for histograms and autocorrelations and are too small to be seen.

Potential sites of interactions between MMP1 and fibrin. MMP3, MMP7, and MMP14 cleave the α-chain at Asp97-Phe98 and Asn102-Asn103; the β-chain at Asp123-Leu124, Asn137-Val138, and Glu141-Tyr142; and the γ-chain at Thr83-Leu84[2] as shown in **Figure S2**.

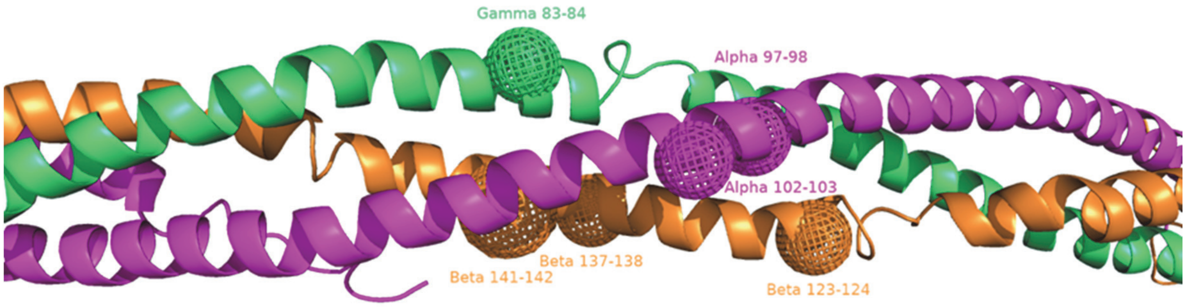

Figure S2. Potential MMP1 cleavage sites on the three fibrin chains. The balls are potential cleavage sites on fibrin that we considered to select the best docking pose between MMP1 and fibrin.

Interdomain distance correlates with the catalytic pocket opening of MMP1 on fibrinogen. In contrast to free MMP1 (**Figure 5**) and fibrin-bound MMP1 (**Figure 6**), fibrinogen-bound

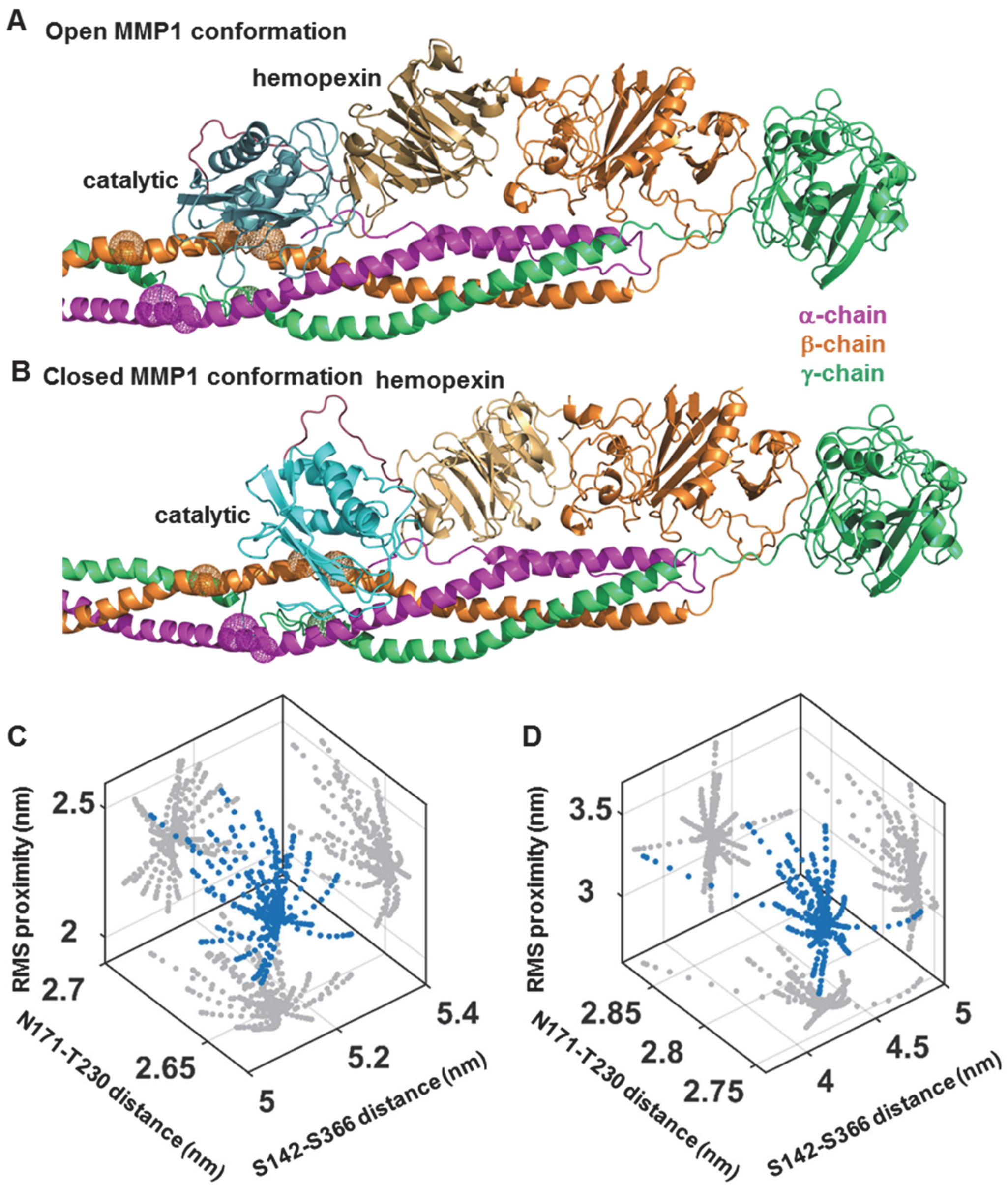

Figure S3. Correlations of MMP1 interdomain distance with catalytic pocket opening when MMP1 is bound to fibrinogen. Examples of (**A**) open and (**B**) closed conformations of MMP1 (PDB ID: 1SU3) bound to fibrinogen (PDB ID: 3GHG). Three dimensional scatter plots (blue circle) of interdomain distance (S142-S366), catalytic pocket opening (N171-T230), and rms proximity between the MMP1 catalytic site and the three fibrinogen chains for (**C**) open and (**D**) closed MMP1 conformations. Two-dimensional projections of the scatter plots are in gray.

MMP1 (**Figure S3**) shows smaller catalytic pocket openings for the open MMP1 conformations. However, fibrinogen-bound MMP1 shows closer proximity to the fibrinogen chains in agreement with free MMP1 and fibrin-bound MMP1. The smaller catalytic pocket opening suggests a different mechanism of fibrinogen and fibrin degradation by MMP1, which needs further studies to confirm.

MMP1 activity on fibrinogen. The point mutation E219Q renders MMP1 catalytically inactive on collagen. For fibrinogen degradation, however, the E219Q mutant degraded the α- and β-chains but did not degrade the γ-chain (**Figure S4A**, lane 4 from left). We used trypsin during MMP1 purification, and trypsin is known to degrade fibrinogen[3]; therefore, any residual trypsin could potentially interfere with the results. We added trypsin inhibitor at 0.5 mg/mL to all the reactions to address this possibility, which was sufficient to inhibit 0.1 mg/mL trypsin (**Figure S4A**, lane 5 from left).

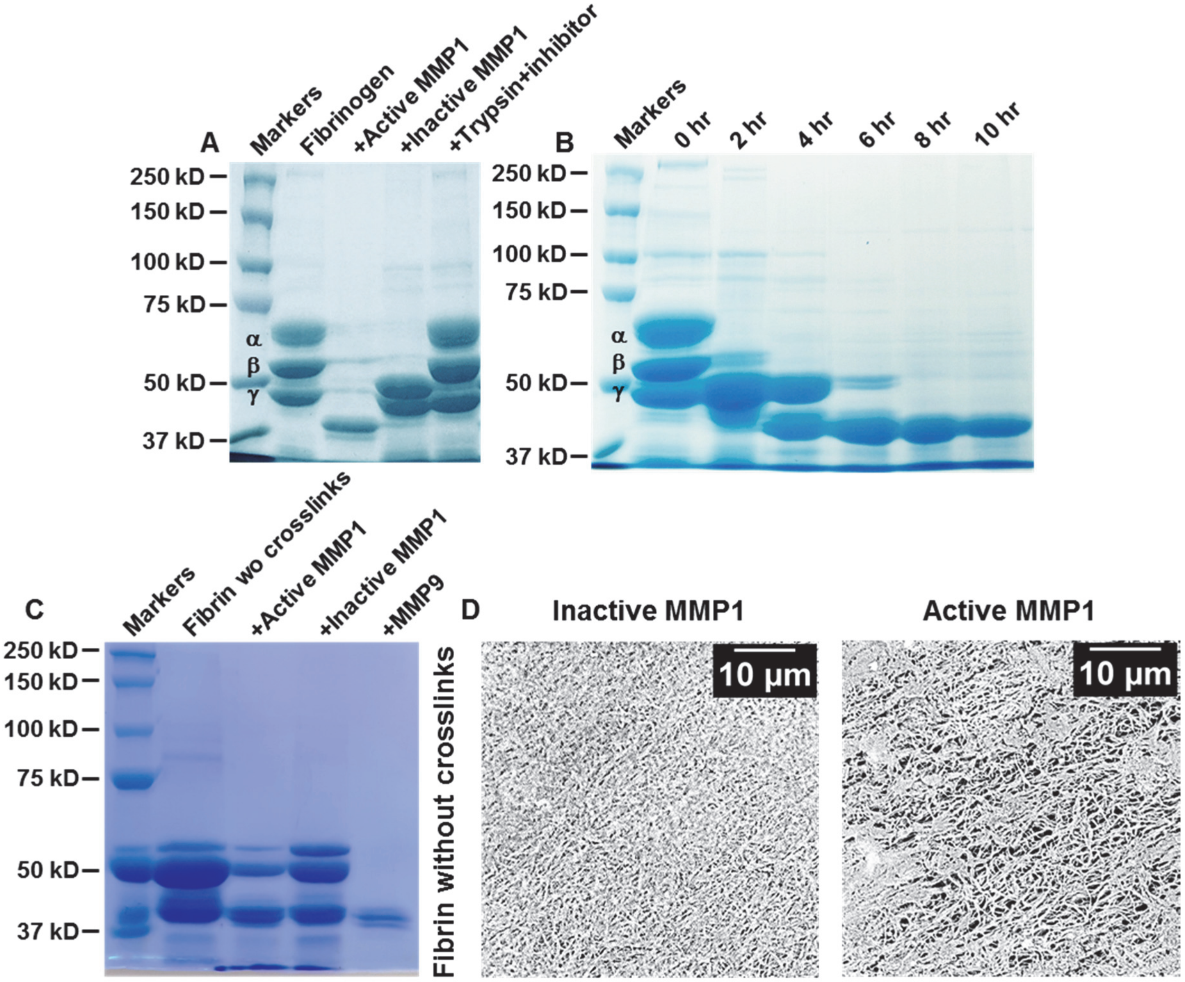

Figure S4. Ensemble activity of MMP1 on fibrinogen and fibrin without crosslinks. (**A**) SDS PAGE of fibrinogen after treatment with different enzymes. Since we used trypsin to activate and purify MMP1, we added 0.5 mg/mL trypsin inhibitor to lane 2-6 to control for any residual effect of trypsin. Trypsin inhibitor (Worthington, Lima Bean, Cat# LS002829) at 0.5 mg/mL inhibited the fibrinolytic effect of 0.1 mg/mL trypsin used in lane 5. Note that the difference between trypsin inhibitor and MMP1 inhibitor (tetracycline). (**B**) Time-dependent fibrinogen degradation by active MMP1. (**C**) SDS PAGE of fibrin without (wo) crosslinks treated with different enzymes. (**D**) SEM images of fibrin after MMP1 treatment. Note that the active site mutant of MMP1 is catalytically inactive on collagen[4]. However, as evident from **Figure S4A**, the active site mutant of MMP1 is partially functional on fibrinogen. In contrast, the active site mutant is catalytically inactive on crosslinked fibrin, as shown in **Figure S5** and **Figure S6**.

Figure S4A does not reveal the sequence in which MMP1 degrades the three chains of fibrinogen. **Figure S4B** shows the time-dependent degradation of fibrinogen. MMP1 degrades the α-chain

first and the γ-chain last. A comparison with prior research shows that plasmin, the well-known fibrinolytic agent, also degrades the α-chain first and the γ-chain last[5]. **Figure S4C** shows SDS PAGE of fibrin without crosslinks treated with different enzymes. As expected, SDS PAGE of fibrin without crosslinks shows the molecular weights of chains lower than fibrinogen (**Figure S4A**). Also, MMP1 degrades the β-chain in fibrin without crosslinks less than in fibrinogen, suggesting a difference of MMP1 interactions with fibrinogen and fibrin without crosslinks. Next, we tested the effects of MMP1 on fibrin morphology using SEM (**Figure S4D**), where the destruction of fibrin structures is visible for active MMP1 compared to inactive MMP1. We created similar thin layers of fibrin morphologies for smFRET experiments. Overall, the ensemble experiments in **Figure S4** show that MMP1 has fibrinolytic activity on fibrinogen and fibrin, and there is a specific sequence of degradation of the three chains. Trypsin cleaves all peptide bonds in fibrinogen that follow arginine or lysine[6].

In comparison, degradation of fibrinogen and crosslinked fibrin by MMPs do not have specific cleavage sites across the MMP-family. For example, MMP3, MMP7, and MMP14 cleave the α-chain at Asp97-Phe98 and Asn102-Asn103; the β-chain at Asp123-Leu124, Asn137-Val138, and Glu141-Tyr142; and the γ-chain at Thr83-Leu84[2]. Cleavage sites on both fibrinogen and crosslinked fibrin for these three MMPs are close to those for plasmin. Additionally, MMP3 solubilizes crosslinked fibrin by cleaving the Gly404-Ala405 bond of the γ-chain[7], resulting in a D-like monomer fragment at ~94 kDa similar to fibrinogen degradation by plasmin. In contrast, MMP7 and MMP14 solubilize crosslinked fibrin and produces D-like dimer fragments at ~186 kDa, similar to crosslinked fibrin degradation by plasmin[2]. However, MMP1, MMP2, MMP9, and MMP15 do not show similar activity[2]. Fibrinogen degradation by catalytic domains of MMP8, MMP12, MMP13, and MMP14 shows differences in the cleavage sites[8]. Overall, MMPs show differences in fibrinolytic activity, which is intriguing because the catalytic domain has a mostly conserved sequence among the MMP-family members[9]. Interestingly, prior research reported insignificant fibrinolytic activity of MMP1[7], in contrast to our findings.

Weight-based degradation assay for water-insoluble fibrin. We used a weight-based degradation assay to quantify the activity of MMP1 because fibrin is water-insoluble, and solution-based biochemical assays are not appropriate. We prepared fibrin with crosslinks by mixing 5 μg of human factor XIII, 20 units of thrombin (Cayman chemical, Cat# 13188), and 40 μL of 10 mM PBS (pH 7.4) in a 0.5 mL PCR tube at 22° C. We incubated the mixture for 10 min at 37° C without shaking. We added 100 μg of fibrinogen (Cayman chemical, Cat# 16088) and 20 μL of 5 mM $CaCl_2$ to the mixture and incubated for an additional 15 min at 37° C without shaking. After incubation, the solution becomes turbid, indicating the formation of crosslinked fibrin. We prepared five reactions for crosslinked fibrin and added 1) 100 μL of 10 mM PBS (pH 7.4), 2) 100 μL of 1 mg/mL active MMP1, 3) 100 μL of 1 mg/mL active MMP1, 4) 100 μL of 1 mg/mL trypsin, and 5) 100 units of thrombin. We made each reaction's final volume 200 μL by diluting with 10 mM PBS (pH 7.4). At 0 hr, we centrifuged the reactions at 10000 rpm for 10 min using a tabletop centrifuge, discarded the supernatant, and weighed the tubes. We subtracted the weights of the PCR tubes for each specific reaction before preparing the reactions. For five conditions, we measured weight of ~120 mg. After weighing, we again added 1) 100 μL of 10 mM PBS (pH 7.4), 2) 100 μL of 1 mg/mL active MMP1, 3) 100 μL of 1 mg/mL active MMP1, 4) 100 μL of 1 mg/mL trypsin, and 5) 100 units of thrombin to a final volume of 200 μL by diluting with 10 mM PBS (pH 7.4). We incubated the reactions at 37° C without shaking. At 1 h, we centrifuged the tubes again, weighed, and subtracted empty tubes' weight. We diluted again as above and incubated for

another hour. We repeated the process at 0 h, 1 h, 2 h, 3 h, 4 h, and 5 h. We performed three biological repeats to calculate the mean and standard deviations. **Figure S5A** shows that active MMP1 and trypsin degrade crosslinked fibrin at a similar rate of ~30 mg/h. The active site mutant of MMP1 and control shows similar weights at a rate of ~7 mg/h. After subtracting the control, the rate of crosslinked fibrin degradation by MMP1 is ~0.23 mg/h/μg. Note that the weight includes water and, as such, has a higher value.

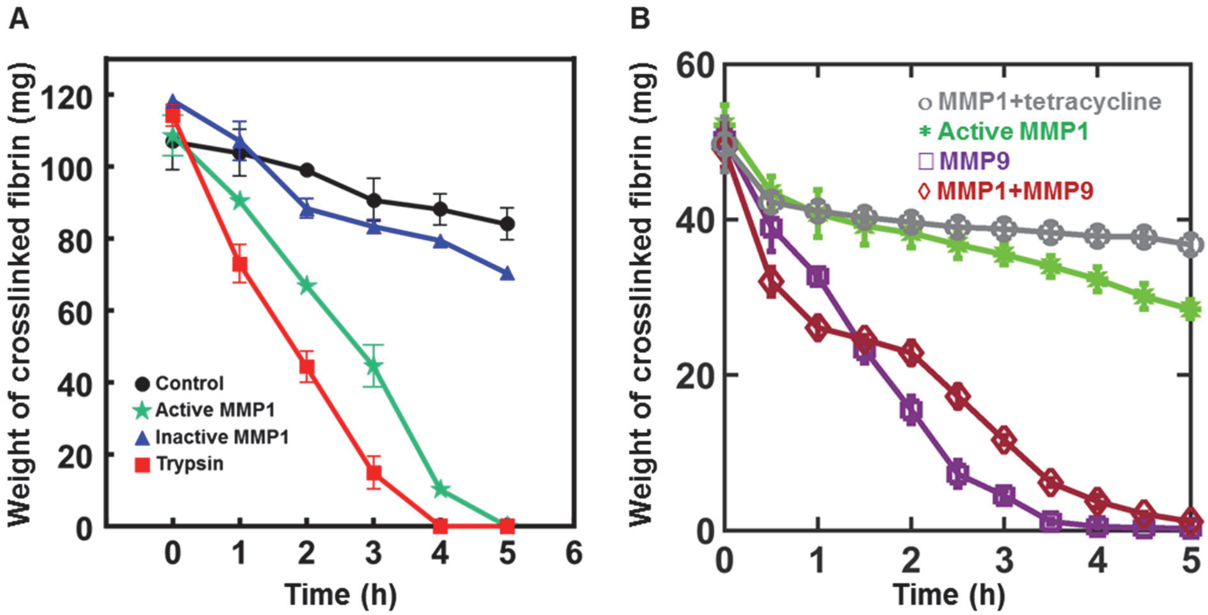

Figure S5. Weight-based fibrin degradation assay. (**A**) Time-dependent weights of crosslinked fibrin after treatment with active MMP1, active site mutant of MMP1, trypsin, and PBS buffer as the control. (**B**) Time-dependent weights of crosslinked fibrin to quantify the effects of tetracycline and MMP9 on fibrin degradation by MMP1. We used 10 times lower concentration of MMP1 and denser fibrin made with 10 times more fibrinogen than (A) to slow down the fibrin degradation.

To resolve the combined effect of MMP1 and MMP9, we prepared fibrin with crosslinks with 10 times higher amount of fibrinogen and used 10 times lower concentration of MMP1 than above. Note that structural rigidity of clot depends on fibrinogen concentrations[10]. We prepared fibrin clots with crosslinks for the weight-based assay in 1.5 mL Eppendorf tubes. We measured the weights of empty tubes for reference, warmed the tubes to 37° C, and added 20 μL of 1000 U/mL thrombin (Cayman chemical, Cat# 13188) and 10 μL of 250 μg/mL Factor XIII (Abcam, Cat# ab62427). Next, we added 40 μL of 25 mg/mL fibrinogen (Cayman chemical, Cat# 16088) to the tubes, followed by 10 μL of 5 mM $CaCl_2$ and 20 μL of 0.01 M PBS. The mixture became cloudy after the addition of fibrinogen and became a gel after the addition of $CaCl_2$. After vortexing briefly, we centrifuged for ~3 s at 10,000 rpm to collect samples at the tube's bottom. We incubated fibrin clot with crosslinks at 37° C without shaking for 15 min. We removed the samples from the incubator and centrifuged at 10,000 rpm for 10 min. After removing excess fluid using 1 mL micropipette tips, we weighed the tubes. The initial weights of clots was calculated by subtracting the empty weight of each individual tube and each clot was treated with a solution containing 1) 100 μL of 0.12 mg/mL active MMP1, 2) 100 μL of 0.12 mg/mL active MMP1 with 0.1 mg/mL tetracycline (Sigma Cat# 87128), 3) 100 μL of 0.14 mg/mL MMP9, and 4) 50 μL of 0.14 mg/mL MMP9 and 50 μL of 0.12 mg/mL active MMP1. After adding solutions containing enzymes, we incubated the reactions at 37° C for 20 min before centrifuging at 10,000 rpm for 10 min. Next,

we removed the excess fluid using 1 mL micropipette tips, weighed the clots, and added back solutions to each clot. We repeated 20 min incubation, followed by 10 min centrifugation and weighing over 5 h with 3 replicates for each sample. **Figure S5B** shows that the combination of MMP1 and MMP9 degrades fibrin faster in the beginning at the rate of ~20 mg/h/μg and then shows the signature of inhibition. MMP9 alone has an activity of ~15 mg/h/μg. We have assumed that fibrin with 10 times higher fibrinogen leads to 10 times slower degradation and adjusted the rate calculated from the data in **Figure S5B** by multiplying with 10. Tetracycline reduces MMP1 activity to a negligible amount, as expected.

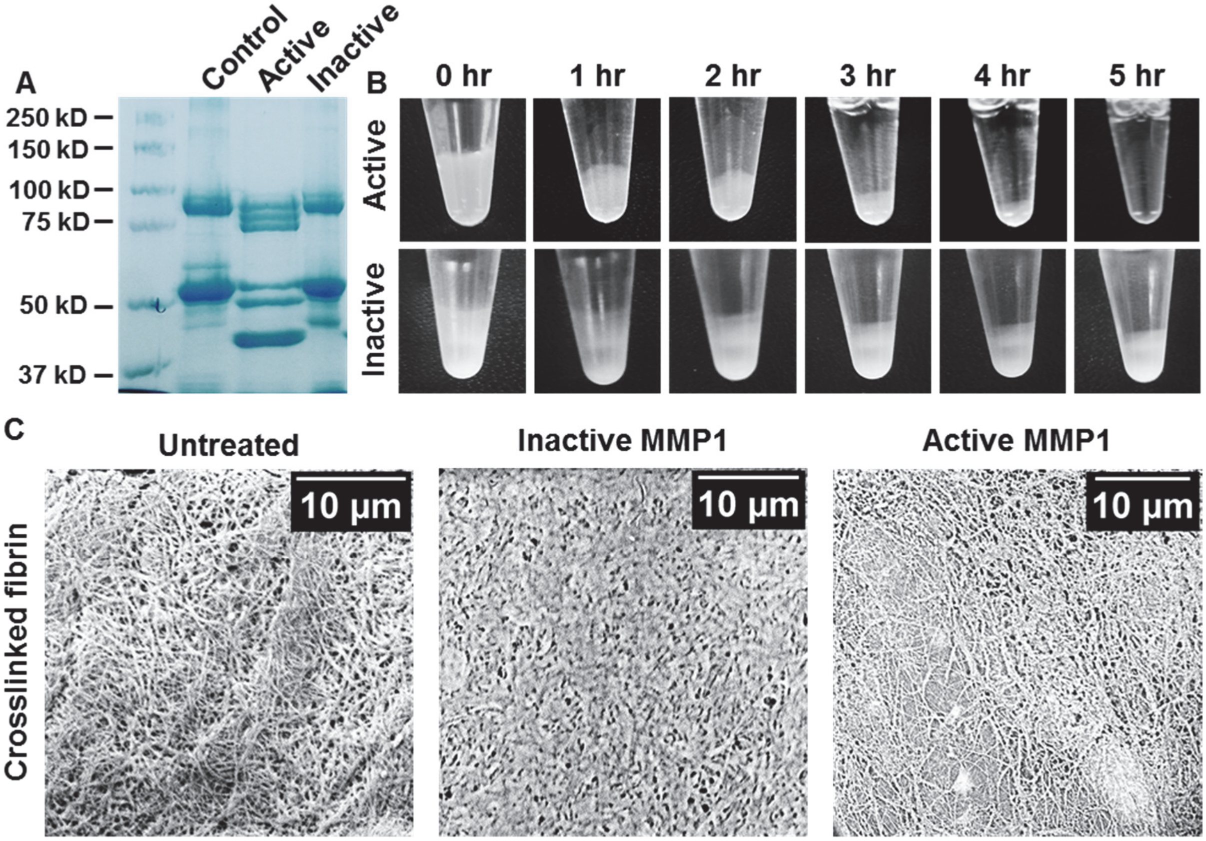

Figure S6. Fibrinolytic activity of MMP1 on crosslinked fibrin. (**A**) SDS PAGE of crosslinked fibrin with active MMP1 and active site mutant of MMP1. The control uses the protein buffer (50 mM Tris, 100 mM NaCl, pH 8.0). (**B**) 100 mg of wet crosslinked fibrin treated with 0.1 mg/mL active MMP1 and active site mutant of MMP1 at 37º C. (**C**) SEM images of crosslinked fibrin with and without MMP1 treatment.

MMP1 activity on crosslinked fibrin. Water-soluble fibrinogen becomes water-insoluble crosslinked fibrin in the presence of factor XIII, thrombin, and $CaCl_2$[11]. Thrombin converts fibrinogen into fibrin monomer by cleaving fibrinopeptide A and fibrinopeptide B[12,13]. Fibrin monomers self-assemble into protofibrils. The polymerization sites noncovalently attach to the D regions of two other fibrin(ogen) molecules. Fibrin monomers in each strand assemble end-to-end, whereas monomers across the strands arrange in a half-staggered overlap. The γ-chains of adjacent D regions in each strand are covalently attached by isopeptide bonds formed due to factor XIIIa and appear as a ~94 kDa band in SDS PAGE under reducing conditions (**Figure S6A**). Since factor XIIIa does not crosslink the β-chains[14,15], the band at ~52 kDa due to the β-chains remains

unchanged in SDS PAGE of fibrinogen (**Figure S6A**) and crosslinked fibrin (**Figure S6A**). As shown in **Figure S6B**, active MMP1 degrades and dissolves water-insoluble crosslinked fibrin, but the active site mutant of MMP1 does not dissolve crosslinked fibrin. **Figure S6C** shows the surface morphology of crosslinked fibrin imaged using SEM. Treatment with active MMP1 resulted in a more porous structure of crosslinked fibrin.

References

1. Arnold, B. C. Pareto distribution. *Wiley StatsRef: Statistics Reference Online*, 1-10 (2014).
2. Bini, A., Wu, D., Schnuer, J. & Kudryk, B. J. Characterization of stromelysin 1 (MMP-3), matrilysin (MMP-7), and membrane type 1 matrix metalloproteinase (MT1-MMP) derived fibrin (ogen) fragments D-dimer and D-like monomer: NH2-terminal sequences of late-stage digest fragments. *Biochemistry* **38**, 13928-13936 (1999).
3. Astrup, T. & Sterndorff, I. Fibrinolytic activity of tissue extracts and of trypsin. *Nature* **170**, 981-981 (1952).
4. Sarkar, S. K., Marmer, B., Goldberg, G. & Neuman, K. C. Single-molecule tracking of collagenase on native type I collagen fibrils reveals degradation mechanism. *Current Biology* **22**, 1047-1056 (2012).
5. Pizzo, S. V., Schwartz, M. L., Hill, R. L. & McKee, P. A. The effect of plasmin on the subunit structure of human fibrinogen. *Journal of biological chemistry* **247**, 636-645 (1972).
6. Andreatta, R. H., Liem, R. K. & Scheraga, H. A. Mechanism of action of thrombin on fibrinogen, I. Synthesis of fibrinogen-like peptides, and their proteolysis by thrombin and trypsin. *Proceedings of the National Academy of Sciences* **68**, 253-256 (1971).
7. Bini, A., Itoh, Y., Kudryk, B. J. & Nagase, H. Degradation of Cross-Linked Fibrin by Matrix Metalloproteinase 3 (Stromelysin 1): Hydrolysis of the γ Gly 404− Ala 405 Peptide Bond. *Biochemistry* **35**, 13056-13063 (1996).
8. Hiller, O., Lichte, A., Oberpichler, A., Kocourek, A. & Tschesche, H. Matrix metalloproteinases collagenase-2, macrophage elastase, collagenase-3, and membrane type 1-matrix metalloproteinase impair clotting by degradation of fibrinogen and factor XII. *Journal of Biological Chemistry* **275**, 33008-33013 (2000).
9. Ratnikov, B. I. *et al.* Basis for substrate recognition and distinction by matrix metalloproteinases. *Proceedings of the National Academy of Sciences* **111**, E4148-E4155 (2014).
10. Ryan, E. A., Mockros, L. F., Weisel, J. W. & Lorand, L. Structural origins of fibrin clot rheology. *Biophysical journal* **77**, 2813-2826 (1999).
11. Weisel, J. W. & Litvinov, R. I. in *Fibrous proteins: structures and mechanisms* 405-456 (Springer, 2017).
12. Medved, L. & Weisel, J. Recommendations for nomenclature on fibrinogen and fibrin. *Journal of Thrombosis and Haemostasis* **7**, 355-359 (2009).
13. Weisel, J. W. & Litvinov, R. I. Mechanisms of fibrin polymerization and clinical implications. *Blood, The Journal of the American Society of Hematology* **121**, 1712-1719 (2013).

14 McKee, P. A., Mattock, P. & Hill, R. L. Subunit structure of human fibrinogen, soluble fibrin, and cross-linked insoluble fibrin. *Proceedings of the National Academy of Sciences* **66**, 738-744 (1970).

15 Sobel, J. H. & Gawinowicz, M. A. Identification of the α chain lysine donor sites involved in factor XIIIa fibrin cross-linking. *Journal of Biological Chemistry* **271**, 19288-19297 (1996).
